# Ligand‐Intercalated MOFs Enable Reaction‐Pathway Engineering in Biomass Electrooxidation via Steric and π‐Electronic Microenvironment Control

**DOI:** 10.1002/anie.4762494

**Published:** 2026-03-27

**Authors:** Junjie Chen, Zhongyuan Guo, Jisheng Xie, Lipeng Tang, Shiyun Li, Yifan Bu, Cheng Peng, Mengyao Zhao, Linda Zhang, Jihan Zhou, Haichao Liu, Hao Li, Mufan Li

**Affiliations:** ^1^ Beijing National Laboratory for Molecular Sciences College of Chemistry and Molecular Engineering Peking University Beijing China; ^2^ Advanced Institute for Materials Research (WPI‐AIMR) Tohoku University Sendai Japan; ^3^ College of Environmental and Resource Sciences Zhejiang University Hangzhou China; ^4^ Frontier Research Institute for Interdisciplinary Sciences Tohoku University Sendai Japan

**Keywords:** biomass electrooxidation, ligand modulation, MOFs

## Abstract

Controlling reaction pathways in electrocatalytic biomass upgrading remains challenging because mass transport, substrate adsorption, and elementary kinetics are intrinsically coupled within catalyst architectures. Here, we report a ligand‐intercalation strategy that enables selective reaction‐pathway engineering in layered metal–organic frameworks (MOFs) by decoupling effects of steric and electronic microenvironments. Aromatic dicarboxylate ligands with systematically varied length and π‐electron density are intercalated into NiCo‐based MOFs to create tunable interlayer nanochannels that independently regulate molecular diffusion and substrate–catalyst interactions. Expanded interlayer spacing enhances alcohol oxidation by improving mass transport and active‐site accessibility, whereas π‐electron‐rich ligands selectively promote aldehyde oxidation through strengthened π–π interactions and accelerated hydrogen atom transfer (HAT), resulting in a shift of the rate‐determining step (RDS) from a chemical to an electrochemical step. These orthogonal effects are quantitatively correlated with kinetic analysis, impedance spectroscopy, adsorption measurements, in situ spectroscopy, and density functional theory calculations. As a result, the optimized MOFs deliver low onset potentials, current densities up to 200 mA cm^−2^, and near‐quantitative Faradaic efficiencies and product yields in the selective oxidation of representative biomass substrates, 5‐hydroxymethylfurfural and 2,5‐diformylfuran. This work establishes ligand‐intercalated MOFs as a versatile platform for microenvironment‐driven reaction‐pathway control in electrocatalytic biomass valorization.

## Graphical abstract

Through ligand length and π‐electron density modulation, metal–organic frameworks (MOFs) improve mass transport and hydrogen atom transfer (HAT), thus improving biomass electrooxidation.

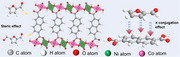

## 1 Introduction

Electrocatalytic biomass upgrading offers a compelling route to couple renewable electricity with sustainable chemical production [1, 2], yet its broader implementation is limited by the difficulty of simultaneously optimizing mass transport [3, 4], substrate adsorption [5], and elementary reaction kinetics [6, 7] within a single catalyst architecture. In many biomass electrooxidation reactions, especially those involving multifunctional oxygenates, catalytic performance is not dictated solely by the intrinsic activity of metal sites but by how effectively reactants access these sites and traverse competing reaction pathways [8]. Achieving deliberate control over such pathway‐defining steps, remains a central challenge in electrocatalyst design.

Metal–organic frameworks (MOFs) provide a uniquely modular platform for addressing this challenge because their catalytic behavior can be tuned not only through metal nodes but also through the spatial and electronic properties of organic ligands. While prior studies have extensively exploited metal doping [9, 10, 11], defect engineering [12, 13, 14], and coordination environment modulation [15] to enhance electrocatalytic activity, the role of organic ligands has largely been confined to structural stabilization or electronic insulation. Much less explored is the possibility of using ligands as active microenvironment regulators that directly govern mass transport, substrate adsorption, and RDSs through steric and non‐covalent interactions.

Layered MOFs are particularly attractive in this context because their interlayer galleries can be systematically expanded or electronically tailored through ligand intercalation [16, 17, 18, 19, 20]. Such architectures offer an opportunity to engineer confined catalytic microenvironments analogous to enzymatic pockets, where steric accessibility and electronic interactions cooperatively dictate reaction pathways. We hypothesized that by judiciously varying ligand length and π‐electron density, it should be possible to decouple steric diffusion effects from electronic adsorption effects, thereby selectively steering biomass electrooxidation pathways at the molecular level.

Here, we examine this hypothesis using a family of layered NiCo‐MOFs intercalated with aromatic dicarboxylate ligands of increasing length and π‐conjugation, including benzenedicarboxylate, naphthalenedicarboxylate, and biphenyldicarboxylate. This design enables independent control over interlayer spacing and π‐electronic character while preserving identical metal coordination environments and electrochemically accessible active‐site densities. Through a combination of kinetic analysis, impedance spectroscopy, adsorption measurements, spectroscopic characterization, and density functional theory calculations, we demonstrate that ligand length governs alcohol oxidation by regulating steric diffusion and active‐site accessibility, whereas ligand π‐electron density selectively enhances aldehyde oxidation by strengthening π–π interactions and accelerating hydrogen atom transfer (HAT), resulting in a shift of the RDS.

Using 5‐hydroxymethylfurfural and 2,5‐diformylfuran as representative biomass substrates, we show that this ligand‐intercalation strategy enables exceptional electrocatalytic performance, including ultrahigh current densities, near‐quantitative Faradaic efficiencies, and outstanding selectivity toward 2,5‐furandicarboxylic acid. More broadly, this work establishes ligand‐intercalated MOFs as a general platform for reaction‐pathway engineering in electrocatalysis, offering a new paradigm for microenvironment‐driven control of complex biomass transformations.

## 2 Results and Discussion

### 2.1 Synthesis and Characterization of NiCo‐Based MOFs

**FIGURE 1:**
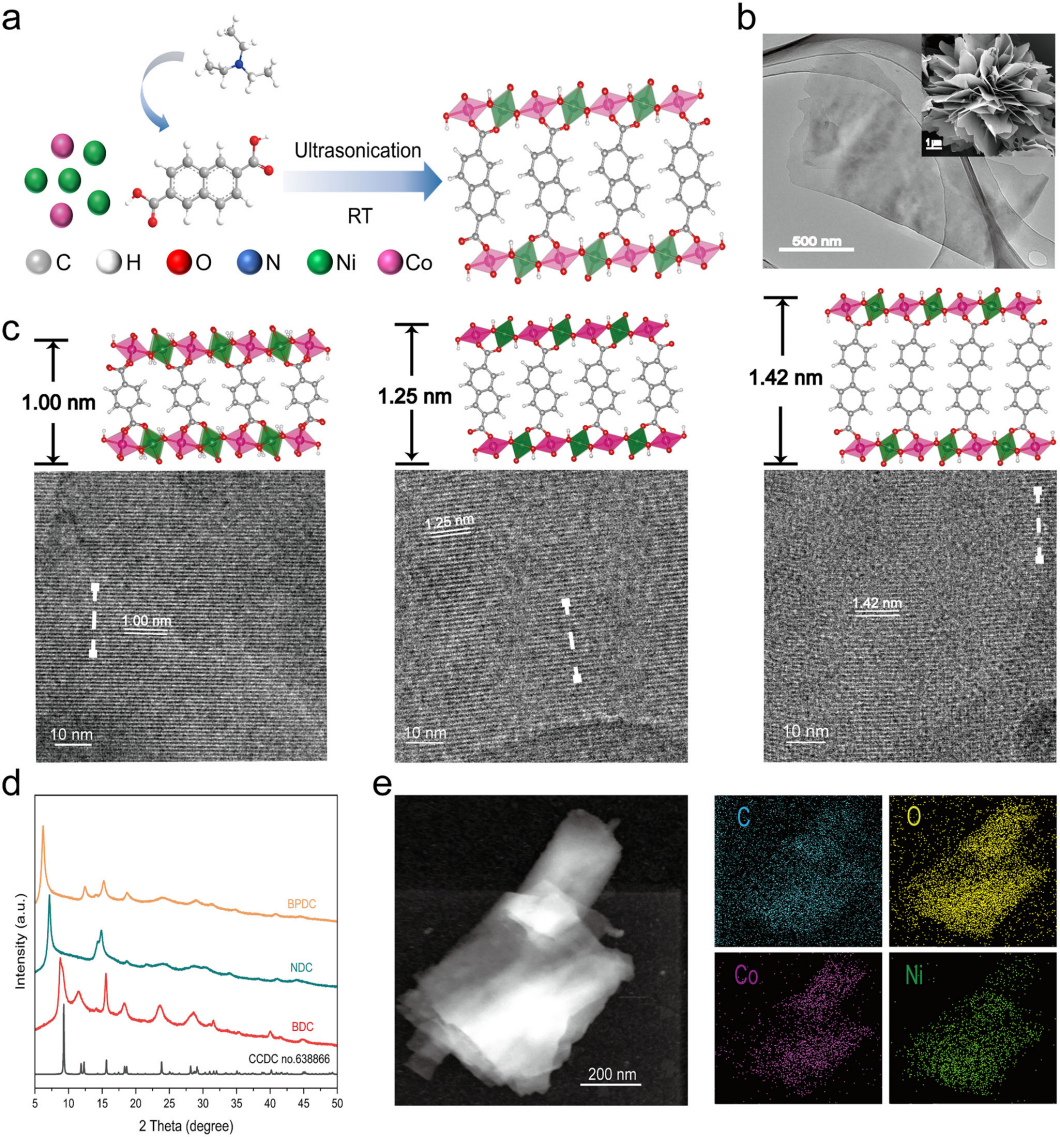
Morphology and structure characterizations of organic ligands intercalated MOFs. (a) Schematic diagram of catalysts synthesis. (b) TEM image of BDC‐MOF. Inset, SEM image. (c) HR‐TEM images of BDC‐MOF. (d) XRD. (e) STEM image and element mapping images of BDC‐MOF.

Three MOFs were synthesized via a simple sonication method from a mixed homogenous solution of Ni^2+^, Co^2+^, and different aromatic organic ligands (Figure 1a). During synthesis, NiCo hydroxide layers formed, and aromatic organic ligands intercalated between layers. Powder x‐ray diffraction patterns (PXRD) analysis confirmed successful intercalation for BDC‐MOF exhibited a crystal phase similar to the Ni‐based reference [Ni_3_(OH)_2_(1,4‐BDC)_2_(H2O)_4_], with Ni/Co ratios of 2.02/1 according to ICP‐AES analysis (Table S1), indicating partial Co substitution. NDC and BPDC‐MOF shared analogous structures but featured larger interlayer spacings, because of larger intercalated organic ligands. Specifically, the first diffraction peak for BDC‐MOF was at 8.7°, indicating the layer distance was about 10.0 Å. However, NDC and BPDC‐MOF with organic ligands of increasing lengths were shown to have the first diffraction peak shifted to lower angles, about 7.2° and 6.2°, corresponding a layer distance of 12.4 and 14.2 Å, respectively. Scanning electron microscopy (SEM) and transmission electron microscopy (TEM) revealed uniform nanosheet morphologies for all MOFs (Figures 1b and S1). The interlayer spacing of three MOFs was also confirmed by High‐resolution transmission electron microscopy (Figure 1c). Line scanning (Figure S2) showed the average lattice fringe spacings of BDC, NDC, and BPDC‐MOF are 10.0, 12.4, and 14.2 Å, respectively, in agreement with the PXRD results. Energy‐dispersive x‐ray spectroscopy (EDS) and high‐angle annular dark‐field scanning transmission electron microscopy (HAADF‐STEM) showed homogeneous distribution of Ni^2+^ and Co^2+^ (Figures 1e, and S3 and S4), confirming that ligand variation had no impact on metal node dispersion.

The effect of organic ligands on the valence state of MOFs metal nodes was further confirmed by x‐ray photoelectron spectroscopy (XPS) (Figure S5b) and Ni K‐edge x‐ray absorption near‐edge structure (XANES) results (Figure S5a). The characteristic peaks of 2p_3/2_ and 2p_1/2_ of Ni in all three MOFs were nearly identical, and the ratio between Ni^2+^ and Ni^3+^ was almost the same to be 2, indicating no obvious valence state difference of Ni was introduced by organic ligands, and the average Ni oxidation state was between 2+ and 3+. This conclusion could also be revealed by Ni K‐edge XANES; no obvious adsorption edge energy shift was observed upon changing different organic ligands in MOFs, and their adsorption position was located more positively compared with Ni foil and NiO, being in agreement with Ni 2p XPS analysis. XPS and XANES data indicated the same Ni valence state in all MOFs.

Extended x‐ray adsorption fine structure (EXAFS) was used to investigate the local coordination environment of Ni in MOFs with different organic ligands at the atomic level. The Fourier transforms of the extended x‐ray adsorption fine structure oscillation profiles (Figure S6) confirmed an identical first shell Ni‐O peak at ∼1.60 Å, which was nearly the same as that of NiO at ∼1.63 Å, and no characteristic peak for Ni‐Ni or Ni‐O‐Ni scatterings compared with Ni foil and NiO, indicating an unchanged Ni local coordination environment in all MOFs.

**FIGURE 2:**
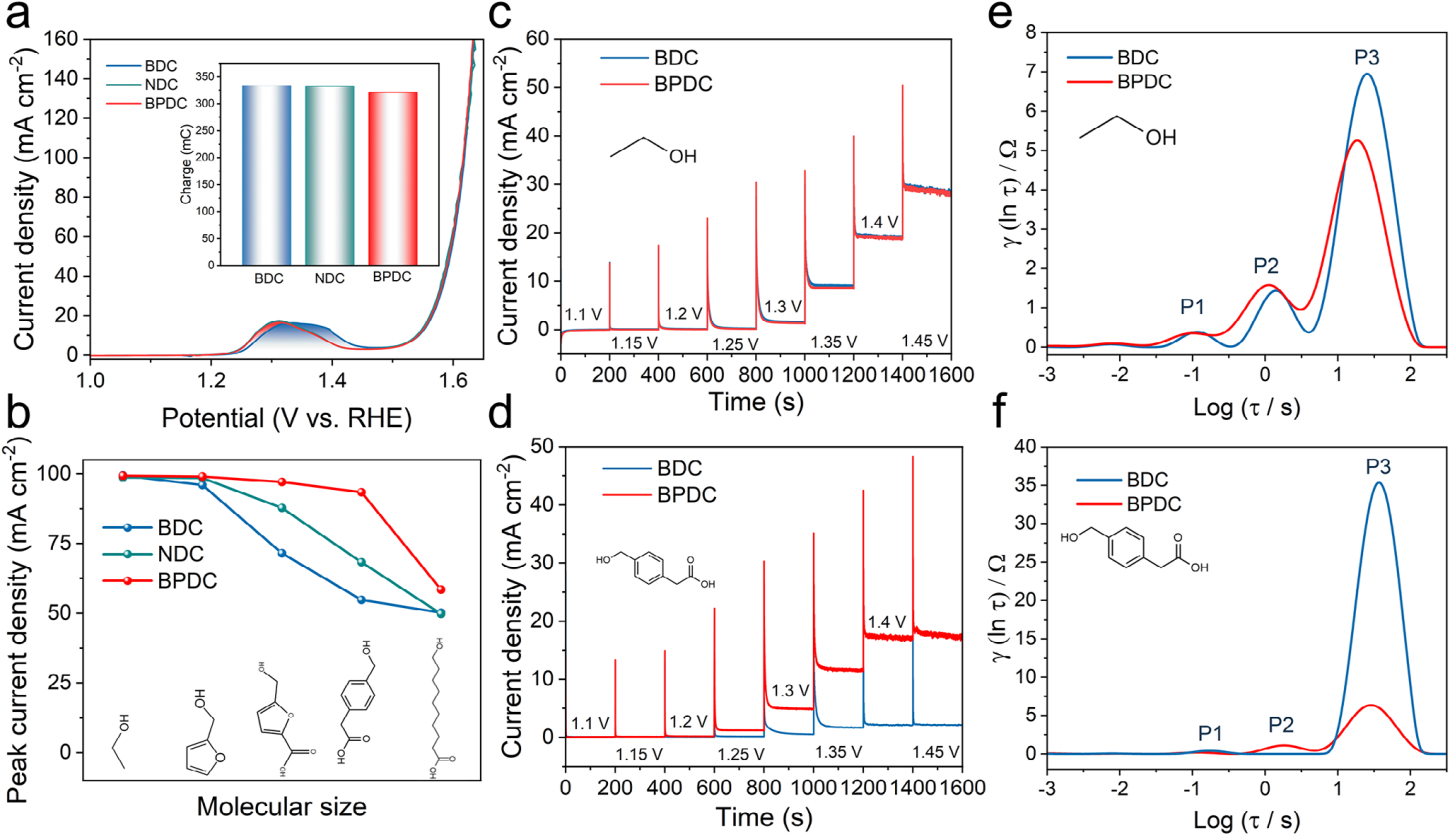
(a) LSV of Ni^2+^ redox peak. Inset, peak area. (b) Peak current density for different alcohols. (c, d) *I*–*t* curves of BDC and BPDC‐MOF at different potentials (V vs. RHE) for ethanol and 4‐(hydroxymethyl) phenylacetic acid, respectively. (e, f) DRT analysis for ethanol and 4‐(hydroxymethyl) phenylacetic acid electrooxidation.

The electrochemical active surface area was compared by measuring Ni^2+^/Ni^3+^ redox area in the LSV curve (Figure 2a) by a three‐electrode configuration in 1 M KOH to determine organic ligand's impact on the number of electrochemical accessible active Ni sites. According to the LSV curve, the oxidation onset potential of Ni^2+^ was about 1.21 V for all three MOFs and ended at 1.45 V for NDC and BPDC, 1.47 V for BDC. Compared with conventional Ni^2+^ electrooxidation onset potential of 1.38 V (Figure S7a), the low onset potential of NiCo‐based MOFs benefited from the incorporation of Co, inducing a higher valence state of Ni and making it easier to be oxidized, as confirmed by XPS of Ni with and without Co incorporation (Figure S7b). An entire Ni^2+^ electrooxidation peak was completely before OER, making it possible to quantify active Ni^2+^ sites by integrating the peak area of Ni^2+^ electrooxidation. The result showed that all MOFs almost had the same number of active Ni sites. Moreover, as the catalyst Ni's mass loading of the electrode was nearly the same as the measured accessible active Ni sites, it could be concluded that almost all Ni sites of the layered MOFs were all accessible for H_2_O molecules and could be electro‐oxidized into Ni^3+^, whether they were on the surface or in the interlayer.

Further, to illustrate the influence of cobalt on the biomass electrooxidation activity, we synthesized Co‐BDC‐MOF as a comparison and took HMFOR as model reaction. As shown in Figure S8, for the LSV curve of OER, Co‐BDC‐MOF exhibited no obvious redox peak, while NiCo‐BDC‐MOF showed crystal Ni^2+^/Ni^3+^ redox peak between 1.21 and 1.47 V. In the presence of 50 mM HMF, NiCo‐BDC‐MOF exhibited a high current density of 125 mA cm^−2^ at 1.4 V. As a comparison, Co‐BDC‐MOF nearly exhibited no activity for the electrooxidation of HMF below 10 mA cm^−2^ before 1.4 V. Clearly, the Ni sites catalyzed the biomass electrooxidation, and the incorporation of Co was mainly to regulate the electronic state of Ni site although Co itself had no catalytic activity at low potentials.

### 2.2 Steric Effect on Electrooxidation of Alcohols

The tunability of ligands with varying lengths enables continuous adjustment of the interlayer spacing in layered MOFs, thereby selectively facilitating the diffusion of substrates with different molecular sizes into the interlayer, enhancing interaction with the electrochemically active Ni sites, and improving both substrate diffusion and accessibility to active sites.

To validate this hypothesis, we compared electrocatalytic oxidation activities of alcohols with different sizes on three MOFs. Ethanol, furfuryl alcohol, 5‐hydroxymethyl‐furan‐2‐carboxylic acid, 4‐(hydroxymethyl) phenylacetic acid, and 10‐hydroxydecanoic acid were chosen. The carboxyl group at the terminus of the longer‐chain alcohols ensured complete dissolution in electrolyte, eliminating potential solubility effects. As shown in Figures 2b and S9, BDC, NDC, and BPDC‐MOF all exhibited high activity (∼100 mA cm^−2^) toward the oxidation of ethanol and furfuryl alcohol. However, as the alcohol size increased, the activity of BDC‐MOF decreased significantly to 71.5 mA cm^−2^ for 5‐hydroxymethyl‐furan‐2‐carboxylic acid, while NDC‐MOF declined to 87.8 mA cm^−2^. In contrast, BPDC‐MOF maintained nearly unchanged activity (97.1 mA cm^−2^). With a further increase in substrate size to 4‐(hydroxymethyl) phenylacetic acid, BPDC‐MOF showed only a slight reduction to 93.4 mA cm^−2^, whereas NDC and BDC‐MOF exhibited substantial decreases to 68.3 and 54.8 mA cm^−2^, respectively. When the alcohol size increased markedly, as with 10‐hydroxydecanoic acid, all three MOFs displayed low activity: 58.5, 49.8, and 50.2 mA cm^−2^ for BPDC, NDC, and BDC‐MOF, respectively, approximately half of their ethanol electrooxidation activity.

Given that MOFs with different organic ligands exhibited nearly identical Ni valence state, coordination environments, and electrochemical active surface areas as proven above, the change in alcohol electrooxidation activity was likely attributable to the organic ligands rather than the metal nodes. The activity exhibited strong dependence on both alcohol molecular size and ligand length. As revealed by the PXRD pattern and HR‐TEM, the interlayer spacing of BDC, NDC, and BPDC‐MOF were 10.0, 12.4, and 14.2 Å, respectively, increasing with ligand length. Since ethanol has a molecular size of approximately 4 Å, it was small enough to readily diffuse into the interlayer of the three MOFs, allowing access to both surface and interlayer Ni^3+^ active sites. This was consistent with the Ni^2+^ to Ni^3+^ electrooxidation behavior observed across all MOFs. Thus, all three MOFs exhibited high and comparable activity for ethanol oxidation. With increasing alcohol size, however, diffusion into the interlayers of BDC or NDC‐MOF became hindered due to their smaller interlayer spacings. In contrast, BPDC‐MOF, with its larger interlayer spacing (14.2 Å), facilitated easier diffusion, providing access to more Ni^3+^ active sites. Consequently, BPDC‐MOF significantly outperformed BDC and NDC‐MOF in the electrooxidation of larger alcohols. When the alcohol was too large for any MOF interlayer, such as 10‐hydroxydecanoic acid, only surface active sites were accessible, resulting in uniformly low activity across all three MOFs.

The sequential chronoamperometric technique was conducted (Figure 2d) to further elucidate steric effect. For ethanol electrooxidation, both BDC and BPDC‐MOF show negligible steady‐state current density below 1.2 V, with a continuous increase from 1.2 to 1.45 V without reaching a plateau, suggesting sufficient Ni^3+^ active sites and enhanced diffusion for small molecules, where rapid mass transport was achieved and electrochemical polarization dominated the reaction. In contrast, for the electrooxidation of 4‐(hydroxymethyl) phenylacetic acid, BDC‐MOF exhibited a current density that plateaued at 1.3 V, indicating complete consumption of the alcohol at the electrode surface and limited diffusion into the interlayer. As a result, concentration polarization dominated, and mass transport became the RDS. For BPDC‐MOF, the steady‐state current density remained significantly higher and increased without plateauing even at 1.45 V, reflecting improved mass transport and greater active site accessibility due to its larger interlayer spacing.

The steric effect‐induced mass transport was further confirmed by electrochemical impedance spectroscopy with distribution of relaxation times (DRT) analysis [21]. For ethanol electrooxidation, three distinct peaks were observed, and P1 and P2 in the short relaxation time region (high frequency) corresponded to Ni‐MOF oxidation and ethanol oxidation, respectively, while P3 in the long relaxation time region (low frequency region) represented mass transport (Figure 2e). There was no apparent difference between BDC and NDC‐MOF in the DRT analysis for the ethanol electrooxidation, indicating comparable and efficient mass transport for small molecules. However, for the electrooxidation of 4‐(hydroxymethyl) phenylacetic acid, a larger molecule than ethanol, BDC‐MOF exhibited a pronounced mass transport peak (P3), indicating hindered diffusion owing to its narrow interlayer spacing compared with BPDC‐MOF. On the contrary, BPDC‐MOF showed a P3 peak similar to that in ethanol electrooxidation, confirming rapid mass transport enabled by its larger interlayer spacing.

### 2.3 π‐Conjugation Effect on Electrooxidation of Aldehydes

The diverse selectivity of aromatic organic ligands enables MOFs to engage in specific interactions with substrates via π–π stacking. Aromatic ligands with higher π‐electron density, such as NDC compared with BDC or BPDC, are likely to exhibit stronger interactions with aromatic aldehydes. Modulating the π‐electron density of aromatic ligands thus provides a strategy for MOFs to selectively enhance the adsorption of substrates, potentially altering the RDS of the electrochemical reaction.

**FIGURE 3:**
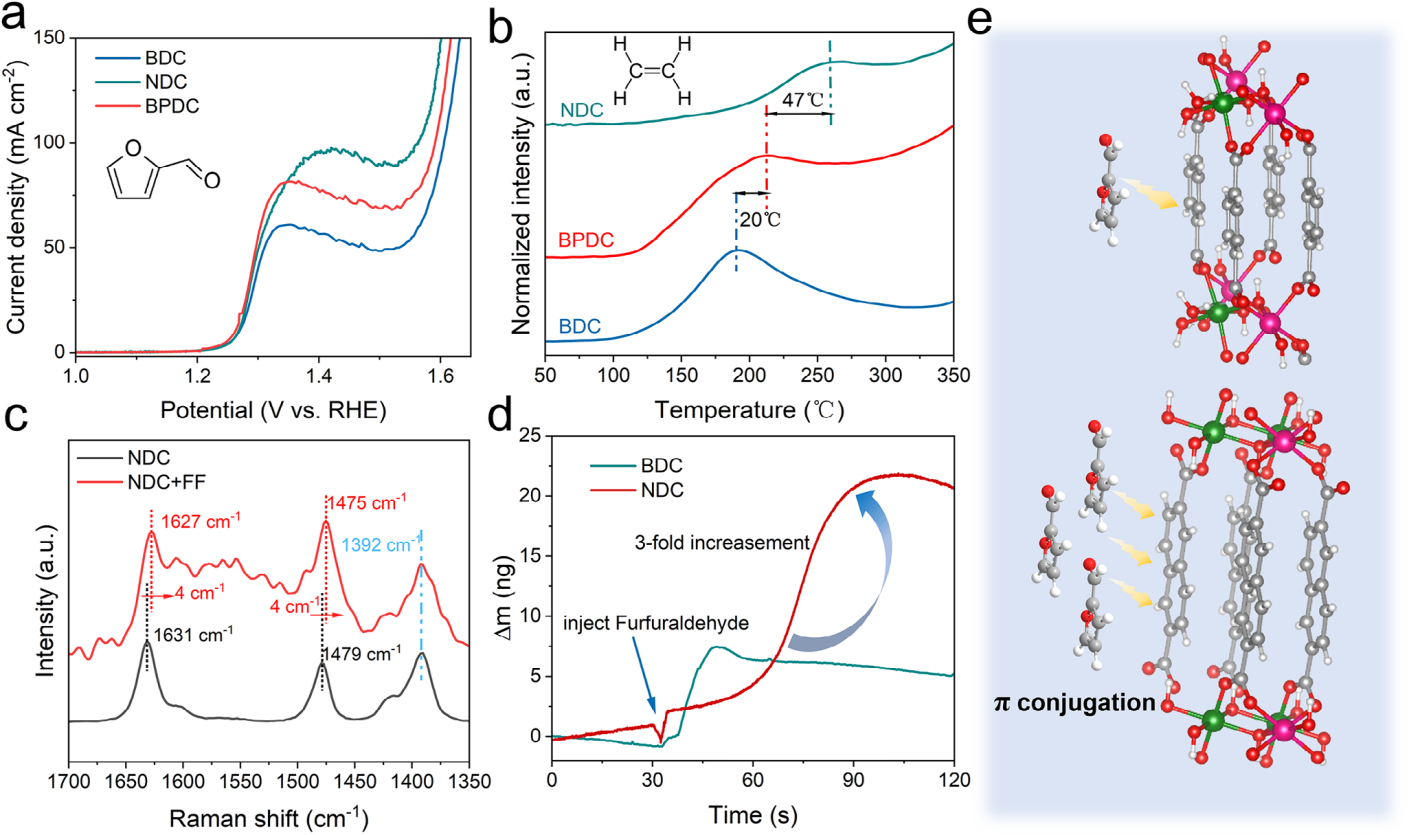
(a) LSV curves of furaldehyde. (b) TPD spectra of ethylene on MOFs. (c) Raman spectra of NDC‐MOF with and without 2‐furaldehyde adsorption. (d) QCM results of 2‐furaldehyde for BDC and NDC‐MOF. (e) Schematic diagram of π‐conjugation effect.

To investigate this effect, the electrooxidation of 2‐furaldehyde was evaluated. As shown in Figure 3a, NDC‐MOF exhibited the highest activity (97.6 mA cm^−2^), followed by BPDC‐MOF (81.6 mA cm^−2^) and BDC‐MOF (60.5 mA cm^−2^). This trend contrasted with the electrooxidation of furfuryl alcohol, where all three MOFs exhibited comparable activities. Given the comparable molecular sizes of 2‐furaldehyde and furfuryl alcohol, the observed differences in electrooxidation performance were most likely attributable to factors other than steric effect. Considering NDC possessed a much higher π‐electron density than BPDC and BDC [20], NDC‐MOF was expected to enhance aldehyde adsorption through π–π interactions, thereby promoting electrocatalytic activity.

Temperature‐programmed desorption (TPD) measurements were conducted to investigate the 2‐furaldehyde adsorption on BDC, NDC, and BPDC‐MOF (Figure 3b). Ethylene was chosen to mimic 2‐furaldehyde as a probe molecule for their similar π‐conjugated molecular structure. Thermo‐gravimetric analysis (Figure S10) confirmed that all three MOFs remained structurally stable up to approximately 400°C; thus, ethylene‐TPD measurement was performed between 50 °C and 350 °C. NDC‐MOF exhibited a significantly higher ethylene desorption temperature (260°C) than BPDC‐MOF (213°C) and BDC‐MOF (193°C), indicating stronger adsorption for π‐conjugated molecules.

Raman spectroscopy further substantiated the role of π‐conjugation (Figure 3c). Characteristic bands at 1631, 1479, and 1392 cm^−1^ were assigned to C═C stretching, C═C─C modes in naphthalene and C═O stretching in carboxyl groups [22], respectively. Upon adsorption of 2‐furaldehyde, the C═C and C═C─C bands in NDC‐MOF exhibited a red shift of 4 cm^−1^, while the C═O band remained unchanged, indicating a strong interaction between 2‐furaldehyde and naphthalene moiety. This behavior aligned with previous reports [23, 24], showing that planar conjugated systems, such as pyrene, induced red shift of carbon nanotube's D and G Raman spectra for strong π–π interactions. In contrast, no Raman spectral shifts were observed for BDC‐MOF before and after 2‐furaldehyde adsorption (Figure S11), further demonstrating stronger π–π interactions for NDC‐MOF with the furanic ring of furaldehyde than BDC‐MOF.

The quartz crystal microbalance (QCM) analysis was employed to quantify furfuraldehyde adsorption and assess π–π interactions between organic ligands and 2‐furaldehyde (Figure 3d). After injecting 50 mM 2‐furaldehyde into the electrolyte under open circuit potential conditions, both BDC and NDC‐MOF exhibited a positive mass change, revealing spontaneous adsorption of 2‐furaldehyde on MOFs. Notably, NDC‐MOF exhibited a threefold greater mass increase compared with BDC‐MOF, reflecting higher furfuraldehyde uptake and stronger π–π interactions, consistent with TPD and Raman results, as summarized in Figure 3e.

### 2.4 Reaction Mechanism Explorations

**FIGURE 4:**
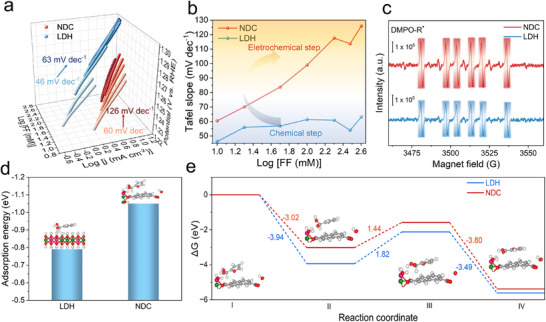
(a,b) Tafel slope of furaldehyde electrooxidation at different concentration. (c) Quasi‐in situ EPR trapping of furaldehyde electrooxidation on NDC‐MOF and LDH. (d) Adsorption energy of diol on LDH and NDC. (e) Reaction pathway for electrooxidation of furfuraldehyde.

The π‐conjugation effect on mechanism was investigated by Tafel slope analysis (Figures 4a,b and S12). The electrooxidation of 2‐furaldehyde contained a couple of sequential electrochemical and chemical elementary steps. First, the electrocatalyst underwent electrochemical dehydrogenation to generate high‐valence Ni species containing electrophilic lattice oxygen. Spontaneously, active Ni species containing electrophilic lattice oxygen captured hydrogen atoms from 2‐furaldehyde to generate divalent Ni species and dehydrogenation products. At low 2‐furaldehyde concentration, the Tafel slope for NDC‐MOF was approximately 60 mV dec^−1^, suggesting the RDS was the second chemical reaction step of HAT process [25, 26, 27, 28], following the initial electrochemical oxidation of Ni^2+^ and generating active NiOOH species, which was in agreement with previous reports on Ni‐based catalysts for aldehydes’ electrooxidation [29, 30, 31]. However, as the 2‐furaldehyde's concentration increased, the Tafel slope gradually rose from 60 to 120 mV dec^−1^, indicating a shift in the RDS to the electrochemical step, specifically the first electron transfer step of Ni's electrooxidation to NiOOH. As a comparison, conventional NiCo layered double hydroxide (LDH) without organic aromatic ligand intercalation maintained a Tafel slope of 60 mV dec^−1^ across all concentrations, indicating the RDS remained the HAT process regardless of concentration. The shift of RDS from a chemical step to an electrochemical step illustrated that strong π–π interactions between 2‐furaldehyde and NDC organic ligand increased the local furaldehyde concentration at the electrode surface, accelerating the chemical HAT process.

To further corroborate the promotion of HAT process in NDC‐MOF, quasi‐in situ electron paramagnetic resonance (EPR) measurements were conducted using 5,5‐dimethyl‐1‐pyrroline‐N‐oxide (DMPO) as the radical trapping agent (Figure 4c). A distinct six‐set peak assigned to the DMPO‐R* was observed for both NDC and LDH catalysts, suggesting the generation of ketyl radical intermediates and the occurrence of the HAT process. Notably, the EPR signal intensity for NDC‐MOF was significantly stronger than that for LDH at the same applied potential, indicating faster reaction kinetics for HAT process attributable to stronger π–π interactions between 2‐furaldehyde and NDC organic ligand.

Theoretical investigations based on first‐principles calculations were further implemented to shed light on the origin of enhanced electrocatalytic activity for furaldehyde oxidation over NDC‐MOF compared to LDH. The adsorption configurations of 2‐furaldehyde on both catalysts were examined. In alkaline electrolyte, the geminal diol form of furaldehyde, resulting from its hydration, was considered as the adsorbate. Theoretical results revealed that diol preferentially adsorbed on the oxidized Ni site via its hydroxyl group (Figure 4d). The adsorption energy (∆*E*_ads_) on NDC‐MOF was −1.05 eV, indicating a highly favorable interaction compared with an ∆*E*_ads_ of −0.79 eV for the adsorption on LDH, underscoring the role of π–π interactions in enhancing adsorption on NDC‐MOF.

In addition, the reaction pathway was further theoretically elucidated (Figure 4e). The reaction initiated with the adsorption of diol on the electrooxidized Ni site. As the potential increased, the adsorbed diol (R‐CH(OH)_2_) underwent a proton–electron coupling transfer process to form R‐CH(O)OH* on a trivalent Ni site. The Ni site was subsequently oxidized to restore the active Ni^3+^ species again. Finally, R‐CH(O)OH* underwent a second proton–electron coupling transfer process, cleaving the C─H bond to yield R‐COOH*. The Gibbs free energy diagrams based on the computational hydrogen electrode method [32, 33] showed that the Gibbs free energy change of the electrooxidation of catalysts to reproduce active Ni sites was the rate‐limiting step with a ∆*G* of 1.44 eV in NDC‐MOF, which is smaller than 1.82 eV in LDH, indicating more favorable reaction kinetics for furaldehyde electrooxidation on NDC‐MOF.

### 2.5 Electrooxidation of HMF and DFF as Model Reactions

**FIGURE 5:**
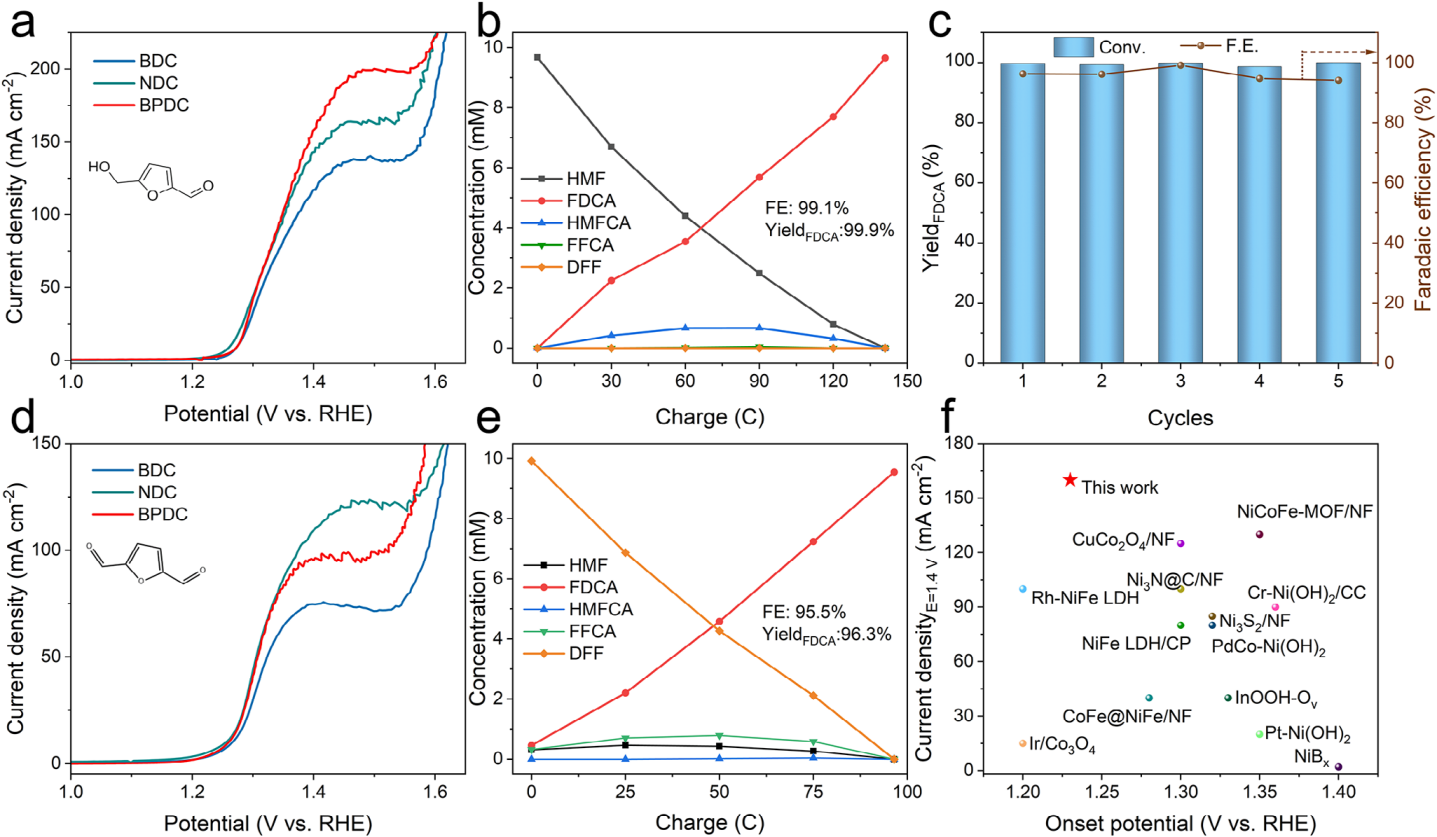
(a, d) LSV curves in 1 M KOH with 0.05 M HMF and 0.05 M DFF, respectively. (b, e) Variation of products with charge in HMFOR and DFFOR catalyzed by BPDC‐MOF and NDC‐MOF, respectively. (c) Plots of FDCA yield and FE for five cycles of HMFOR catalyzed by BPDC‐MOF. (f) Comparison of HMFOR performance of BPDC‐MOF with the recently published electrocatalysts.

The electrocatalytic performance for the oxidation of 5‐hydroxymethylfurfural (HMFOR) was evaluated in a three‐electrode configuration using 1 M KOH electrolyte containing 0.05 M HMF. The linear sweep voltammetry (Figure 5a) revealed that BDC, NDC, and BPDC‐MOF exhibited similar and low onset potential (defined here as the potential at 2 mA cm^−2^) of approximately 1.23 V. However, BPDC‐MOF delivered an ultrahigh current density of 160 mA cm^−2^ at 1.40 V, significantly surpassing those of NDC and BDC‐MOF, which could be attributed to its larger interlayer spacing. This combination of low onset potential and high current density exceeded the performance of most state‐of‐the‐art 3D transition metal‐based catalysts and even could be competitive with several representative noble metal catalysts (Figure 5f). To identify and quantify the oxidation products and determine the corresponding Faradaic efficiency, potential static electrolysis of HMF was conducted at 1.38 V in a divided cell using BPDC‐MOF. As illustrated in Figure S13, HMF oxidation could proceed via different intermediates, such as 2,5‐diformylfuran (DFF) and 5‐hydroxymethyl‐2‐furancarboxylic acid (HMFCA), which were subsequently oxidized to 2‐formyl‐5‐furancarboxylic acid (FFCA) and finally to 2,5‐furandicarboxylic acid (FDCA). The complete oxidation of HMF to FDCA involved a six‐electron transfer process, requiring a theoretical charge of 141 C. High‐performance liquid chromatography (HPLC) was employed to monitor organic species during electrolysis and elucidate the reaction pathway (Figure S14). As shown in Figures 5b and S15, the concentration of HMF decreased while that of FDCA increased with accumulated charge. DFF was nearly undetectable, whereas HMFCA and FFCA were observed as intermediates, indicating a HMFCA intermediate pathway, which had been proved to be favorable in alkaline electrolyte. Notably, the FDCA yield of nearly 100% was achieved with a Faradaic efficiency of 99.1%. The stability of the BPDC‐MOF was assessed over five consecutive electrolysis cycles. The FDCA yield remained at 100%, with a Faradaic efficiency of approximately 94% (Figure 5c), demonstrating excellent electrochemical stability. Post‐electrolysis characterization by XRD and XPS was performed to monitor structural and chemical evolution. The XRD patterns (Figure S17) showed the first diffraction peak was retained, confirming that the interlayer spacing remained unchanged and the BPDC ligand was still intercalated within NiCo hydroxide layers. Similarly, the positions and shapes of Ni 2p XPS peaks (Figure S18) were nearly unchanged, suggesting that the valence state and chemical environment of the catalyst remained stable.

The oxidation of 2,5‐diformylfuran (DFFOR) experiment is similar to HMFOR. The linear sweep voltammetry curves (Figure 5d) showed that all three MOFs exhibited a low onset potential of 1.23 V for DFFOR. NDC‐MOF achieved the highest activity of 110 mA cm^−2^ at 1.40 V, attributed to the higher π‐electron density of NDC ligand and the planer conjugated electronic structure of DFF. Similarly, potential static electrolysis of DFF was performed at 1.38 V to analyze products and determine Faradaic efficiency. Analogous to HMF oxidation, DFF is first oxidized to FFCA and subsequently to FDCA via a four‐electron transfer process, requiring a theoretical charge of 94 C for complete conversion. As shown in Figures 5e and S16, the yield and Faradaic efficiency of FDCA both exceeded 95%, indicating the high activity of the NDC‐MOF catalyst. Furthermore, XRD patterns (Figure S17) and XPS spectra (Figure S18) confirmed that catalyst's interlayer spacing and Ni valence state remained stable after DFFOR electrolysis, indicating excellent structural and chemical stability.

To assess the university and general applicability of steric and π‐conjugation effects in MOF‐catalyzed biomass electrooxidation, we conducted electrocatalytic experiments using BPDC‐MOF for four different alcohols and NDC‐MOF for four distinct aldehydes (Figure S19). The catalytic results demonstrated that both BPDC and NDC‐MOF exhibited high activity, affording the corresponding carboxylic acids with yields and Faradaic efficiencies exceeding 90% for alcohol and aldehyde substrates. These findings reveal that rational modulation of organic ligands, including steric control and π‐conjugation enhancement, provides an effective strategy for improving the electrocatalytic performance of MOFs in biomass upgrading reactions.

## 3 Conclusion

In summary, we demonstrate a ligand‐engineering strategy in layered NiCo–MOFs that enables microenvironment‐driven control of biomass electrooxidation pathways. By intercalating aromatic ligands with systematically varied length and π‐electron density, the interlayer nanochannels can be tailored to independently regulate steric accessibility and electronic substrate–catalyst interactions. Ligand length governs alcohol oxidation by expanding interlayer spacing to enhance molecular diffusion and active‐site accessibility, whereas π‐electron‐rich ligands selectively promote aldehyde oxidation by strengthening π–π interactions, accelerating HAT, and shifting the RDS from a chemical to an electrochemical step. Using 5‐hydroxymethylfurfural and 2,5‐diformylfuran as model substrates, the optimized MOFs exhibit low onset potentials, high current densities, and near‐quantitative Faradaic efficiencies and FDCA yields. More broadly, this work establishes organic ligands as active microenvironment regulators rather than passive structural components, providing a versatile framework for reaction‐pathway engineering in electrocatalytic biomass valorization and related transformations.

## Conflicts of Interest

The authors declare no conflicts of interest.

## Supporting information




**Supporting File 1**: The authors have cited additional references within the Supporting Information. [34–40]

## Data Availability

The data that supports the findings of this study are available in the supporting information of this article
