# Supplementary material for: Ligand‐Intercalated MOFs Enable Reaction‐Pathway Engineering in Biomass Electrooxidation via Steric and π‐Electronic Microenvironment Control
Source: Angew Chem Int Ed Engl. 2026 Mar 27;65(19):e4762494. doi: 10.1002/anie.4762494 (PMC13134599; doi:10.1002/anie.4762494)
Supplement: Supplementary file 1 — Supporting File 1: The authors have cited additional references within the Supporting Information. [34–40] [file ANIE-65-e4762494-s001.docx]

**Ligand-Intercalated MOFs Enable Reaction-Pathway Engineering in Biomass Electrooxidation via Steric and π-Electronic Microenvironment Control**

Junjie Chen^a‡^, Zhongyuan Guo^b,c‡^, Jisheng Xie^a^, Lipeng Tang^a^, Shiyun Li^a^, Yifan Bu^a^, Cheng Peng^a^, Mengyao Zhao^a^, Linda Zhang^b,d^, Jihan Zhou^a^, Haichao Liu^a^,^*^ Hao Li^b^,^*^ and Mufan Li^a*^

^a^ Beijing National Laboratory for Molecular Sciences, College of Chemistry and Molecular Engineering, Peking University, Beijing 100874, China.

^b^ Advanced Institute for Materials Research (WPI-AIMR), Tohoku University, Sendai 980-8577, Japan.

^c^ College of Environmental and Resource Sciences, Zhejiang University, Hangzhou 310058, China.

^d^ Frontier Research Institute for Interdisciplinary Sciences, Tohoku University, Sendai 980-0845, Japan.

^‡^ These authors contributed equally to this work.

^*^ Corresponding authors. E-mails: hcliu@pku.edu.cn; [li.hao.b8@tohoku.ac.jp](mailto:li.hao.b8@tohoku.ac.jp); mufanli@pku.edu.cn

**EXPERIMENTAL DETAILS**

**Synthesis of NiCo-BDC-MOF.** First, *N, N*-dimethylformamide (16 mL), ethanol (1 mL) and deionized water (1 mL) were mixed. Next, 0.75 mmol BDC was dissolved into the mixed solution under sonication. Subsequently, 0.5 mmol NiCl_2_·6H_2_O and 0.25 mmol CoCl_2_·6H_2_O were added. After Ni^2+^ and Co^2+^ salts were dissolved, 0.8 mL triethylamine was quickly injected into the solution. Then, the solution was stirred for 5 min to obtain a uniform colloidal suspension. Afterwards, the colloidal solution was continuously ultrasonicated for 8 h (40 kHz) under airtight conditions. Finally, the products were obtained via centrifugation, washed with ethanol (3~5 times), and dried at room temperature.

**Synthesis of NiCo-NDC-MOF.** The process was similar to the NiCo-BDC-MOF synthesis, except that BDC was replaced by NDC.

**Synthesis of NiCo-BPDC-MOF.** The process was similar to the NiCo-BDC-MOF synthesis, except that BDC was replaced by BPDC and *N, N*-dimethylformamide was replaced by *N, N*-dimethylacetamide to ensure the dissolution of organic ligand.

**Synthesis of Ni-BDC-MOF.** The process was similar to the NiCo-BDC-MOF synthesis, except that 0.5 mmol NiCl_2_·6H_2_O and 0.25 mmol CoCl_2_·6H_2_O were replaced by 0.75 mmol NiCl_2_·6H_2_O.

**Synthesis of Co-BDC-MOF.** The process was similar to the NiCo-BDC-MOF synthesis, except that 0.5 mmol NiCl_2_·6H_2_O and 0.25 mmol CoCl_2_·6H_2_O were replaced by 0.75 mmol CoCl_2_·6H_2_O.

**Synthesis of NiCo LDH.** 5 mmol Ni(NO_3_)_2_·6H_2_O and 2.5 mmol Co(NO_3_)_2_·6H_2_O were dissolved in a mixture solvent of 37.5 mL ethylene glycol and 15 mL deionized water. Then, 37.5 mmol of urea was added under stirring. The resulting solution was vigorous magnetic stirring for 3 h at 90 ℃. Then, the precipitates were filtered and washed several times with distilled water and ethanol and dried at 60 ℃.

**Electrochemical measurements.** Electrochemical tests were carried out on an electrochemical workstation of CHI 760E instruments using a typical H-type three-electrode configuration with graphite carbon rod as the counter electrode and a Hg/HgO (1 M KOH) as the reference electrode. To prepare the working electrode, the investigated catalysts (5 mg), carbon black (5 mg) and Nafion 117 solutions (40 μL) were dispersed in ethanol (960 μL) and ultrasonicated for 30 min to obtain a homogeneous catalyst ink. Then 200 μL ink was cast onto a clean carbon paper and dried in air. N_2_-saturated freshly prepared 1 M KOH with 0.05 M alcohols or aldehyde and 1 M KOH were chosen as the electrolytes for electrooxidation experiments. Polarization curves were recorded at a scan rate of 5 mV s^-1^ at room temperature, unless noted otherwise. All collected potentials (E) were converted into the reversible hydrogen electrode (RHE) using the equation: E (RHE) = E (Hg/HgO) + 0.059 pH + E^0^ (Hg/HgO).

**Product analysis for electrooxidation of HMF and DFF.** The products of electrooxidation of the HMF and DFF were analyzed using a high-performance liquid chromatograph (HPLC, Shimadzu- Prominence Plus LC-20A). 20 μL of the electrolyte product was mixed with 1980 μL 5 mM H_2_SO_4_, and then analyzed it using HPLC. The HPLC instrument was equipped with a photodiode array detector and using a 5 um C18 column with a dimension of 4.6 mm * 250 mm. A mixture of eluting solvents (A and B) were used. Mobile phase A was 5 mM ammonium formate aqueous solution and phase B was methanol (a ratio of A : B was 9 : 1, flow rate was 0.5 mL min^-1^, column temperature was 40 ℃). The identification and quantification of the liquid products were derived from calibration curve through applying standard solution with known concentration of commercially pure reagents.

The yield of FDCA, the selectivity of FDCA and the Faradaic efficiency of FDCA for HMFOR were calculated according to the following equation.

FDCA yield (%) = *n* (FDCA formed) / *n* (HMF initial) * 100 %

FDCA selectivity (%) = *n* (FDCA formed) / *n* (HMF consumed) * 100 %

FDCA yield (%) = *n* (FDCA formed) / [Charge / (6 * F)] * 100 %

The yield of FDCA, the selectivity of FDCA and the Faradaic efficiency of FDCA for DFFOR were calculated according to the following equation.

FDCA yield (%) = *n* (FDCA formed) / *n* (DFF initial) * 100 %

FDCA selectivity (%) = *n* (FDCA formed) / *n* (DFF consumed) * 100 %

FDCA yield (%) = *n* (FDCA formed) / [Charge / (4 * F)] * 100 %

F represents the Faraday constant and *n* is the mol of organic compounds calculated from the concentrations determined by HPLC.

**Structural Characterizations.** TEM measurements were conducted on a FEI Tecnai F20 transmission electron microscopy. SEM measurements were conducted on MERLIN Compact field-emission scanning electron microscope. XRD pattern was collected at PANalytical-XRD instrument equipped with a Cu Kα radiation. XPS spectra were performed on Axis Supra instrument. ICP-AES was conducted on Agilent 8800 instrument.

**XAS measurements.** Ni K-edge spectra were collected using a Lytle detector at 1W1B beamline of the Beijing Synchrotron Radiation Facility (BSRF).

**Computational details.** All spin-polarized calculations were carried out with the Vienna Ab Initio Simulation Package (VASP6). The Projector Augmented Wave (PAW) method was employed to describe electron-ion interactions.^1, 2^ The exchange-correlation effects of electrons were treated with the revised Perdew-Burke-Ernzerhof (RPBE) functional,^3^ a generalized gradient approximation (GGA). The kinetic energy cut-off for the plane-wave basis set was set to 520 eV. A (3×3×1) k-point mesh through the Monkhorst-Pack scheme was used to sample the Brillouin zone.^4^ The dispersion interaction was described by the DFT-D3 scheme.^5, 6^ Due to insufficient consideration of the on-site Columbic repulsion, between Ni-*d* or Co-*d* electrons, the GGA + U approach was employed with U-J = 6.6 and 4.0 eV for the Ni and Co atoms, respectively. All surface slabs were built with the vacuum thickness of at least 15 Å in the *z*-direction to avoid the interaction between the periodic structures. All geometry optimizations were carried out until the residual forces on the system converged below 0.03 eV/Å, with the electronic self-consistent (SC) loop convergence threshold set to 1×10^-5^ eV. Based on the computational hydrogen electrode (CHE) method,^7^ the Gibbs free energy change can be calculated by

∆*G* = ∆*E* + ∆*E*_ZPE_ – *T*·∆*S* + $\int{\Delta C}_{P}(T)dT$

where ∆*E*, ∆*E*_ZPE_, and ∆*S* are the electronic reaction energy, the changes of zero-point energy (ZPE), and entropy (*S*), respectively. $\int C_{P}(T)dT$ is the contribution of heat capacity (*C*_p_). The vibration frequencies were analyzed by the DFT calculations. *T* is the reaction temperature (T = 298.15 K). The *ZPE*, *S*, and $\int C_{P}(T)dT$ could be obtained using the harmonic approximation. The adsorption energy (∆*E*_ads_) was defined by

∆*E*_ads_ = *E*_total_ – *E*_slab_ – *E*_adsorbate_,

Where *E*_total_, *E*_slab_, and *E*_adsorbate_ represent the electronic energies of the slab-adsorbate composite, the catalyst slab, and the free adsorbate itself, respectively.


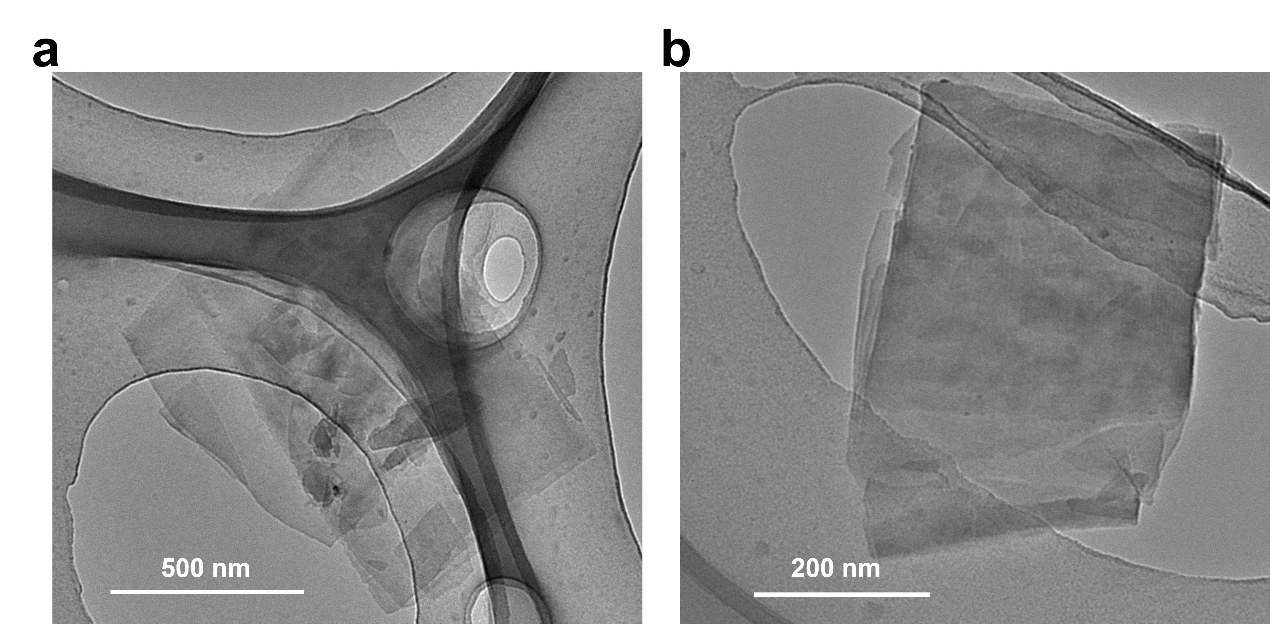


**Figure S1**. TEM image of (a) NDC-MOF and (b) BPDC-MOF.


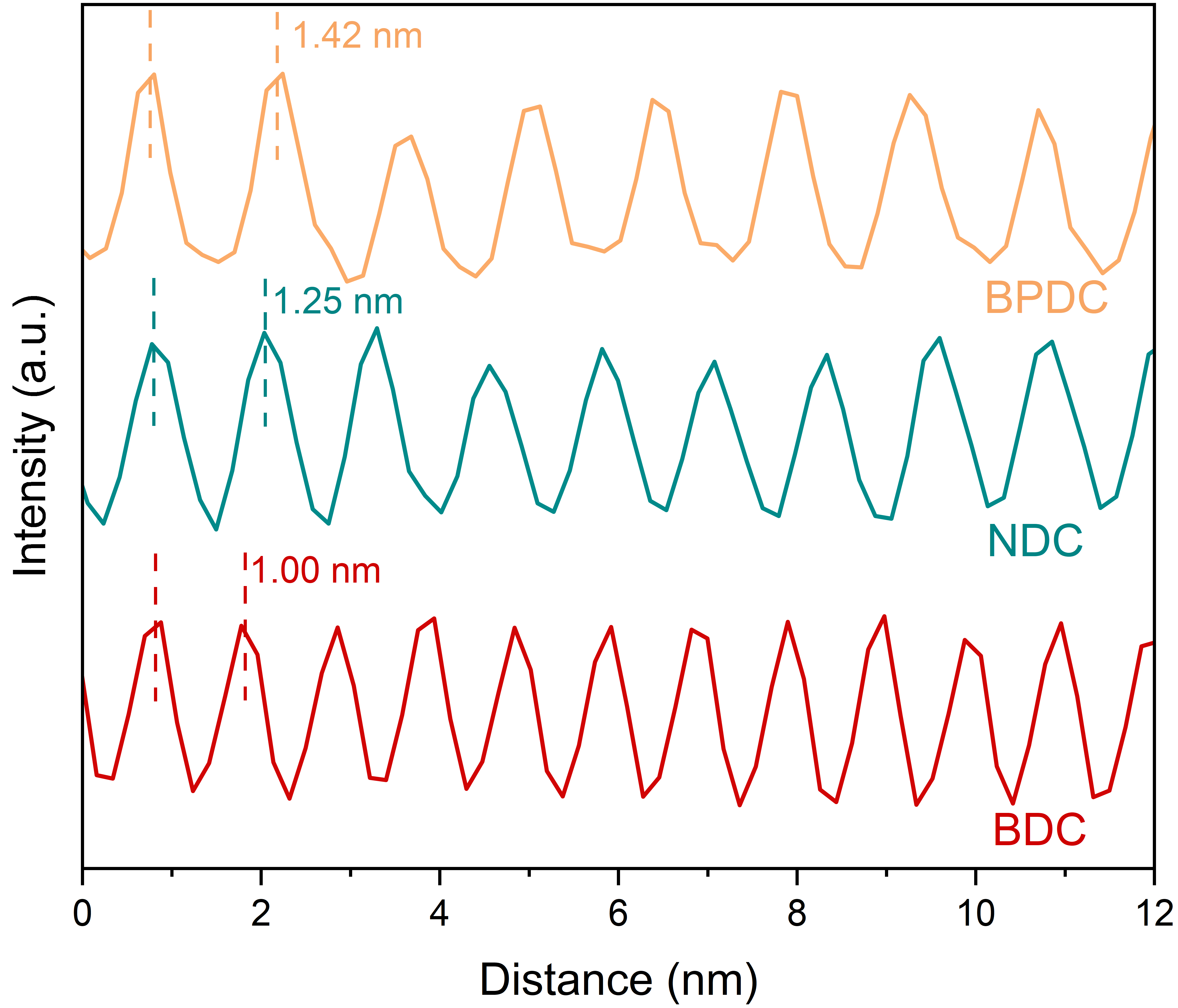


**Figure S2**. Line scanning of three MOFs lattice fringe.


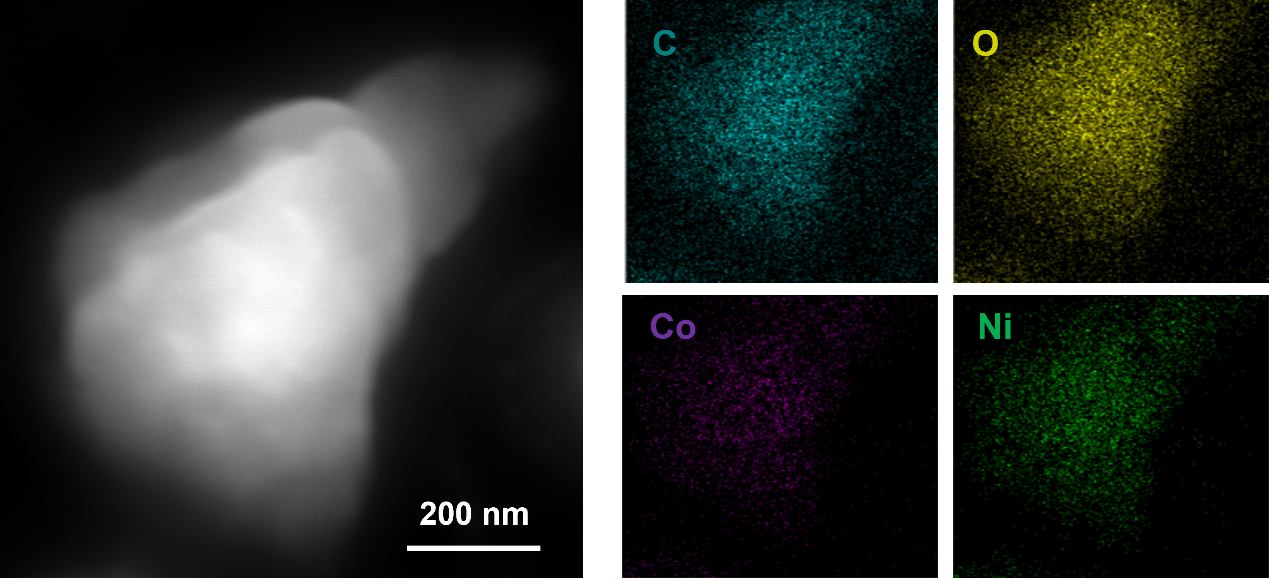


**Figure S3**. STEM image and element mapping images of NDC-MOF.


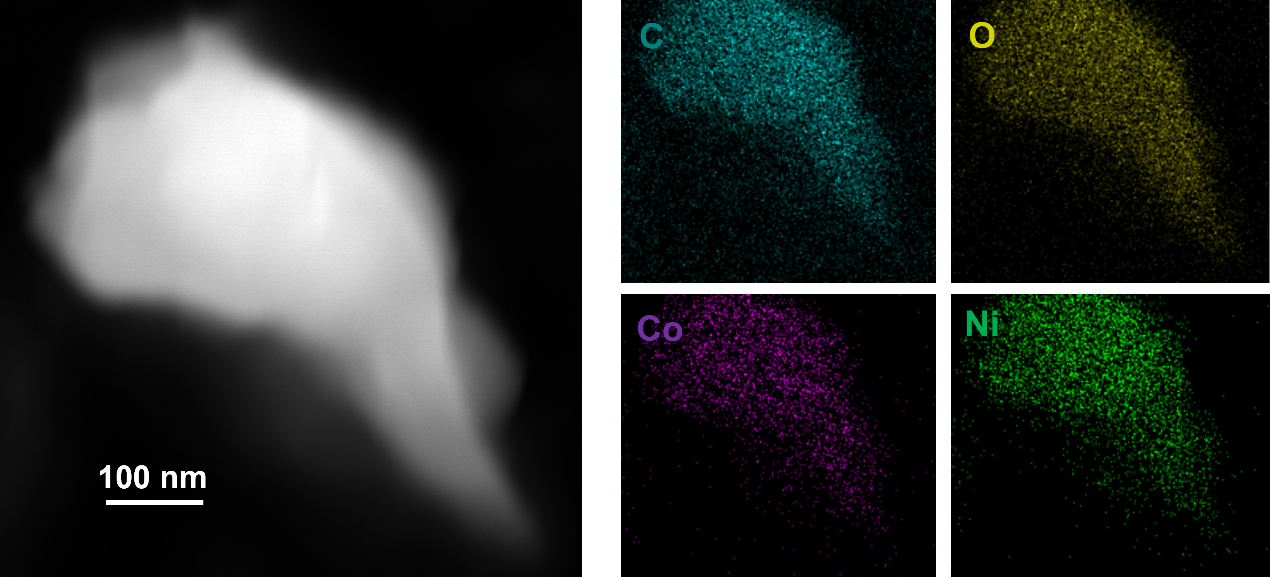


**Figure S4**. STEM image and element mapping images of BPDC-MOF.


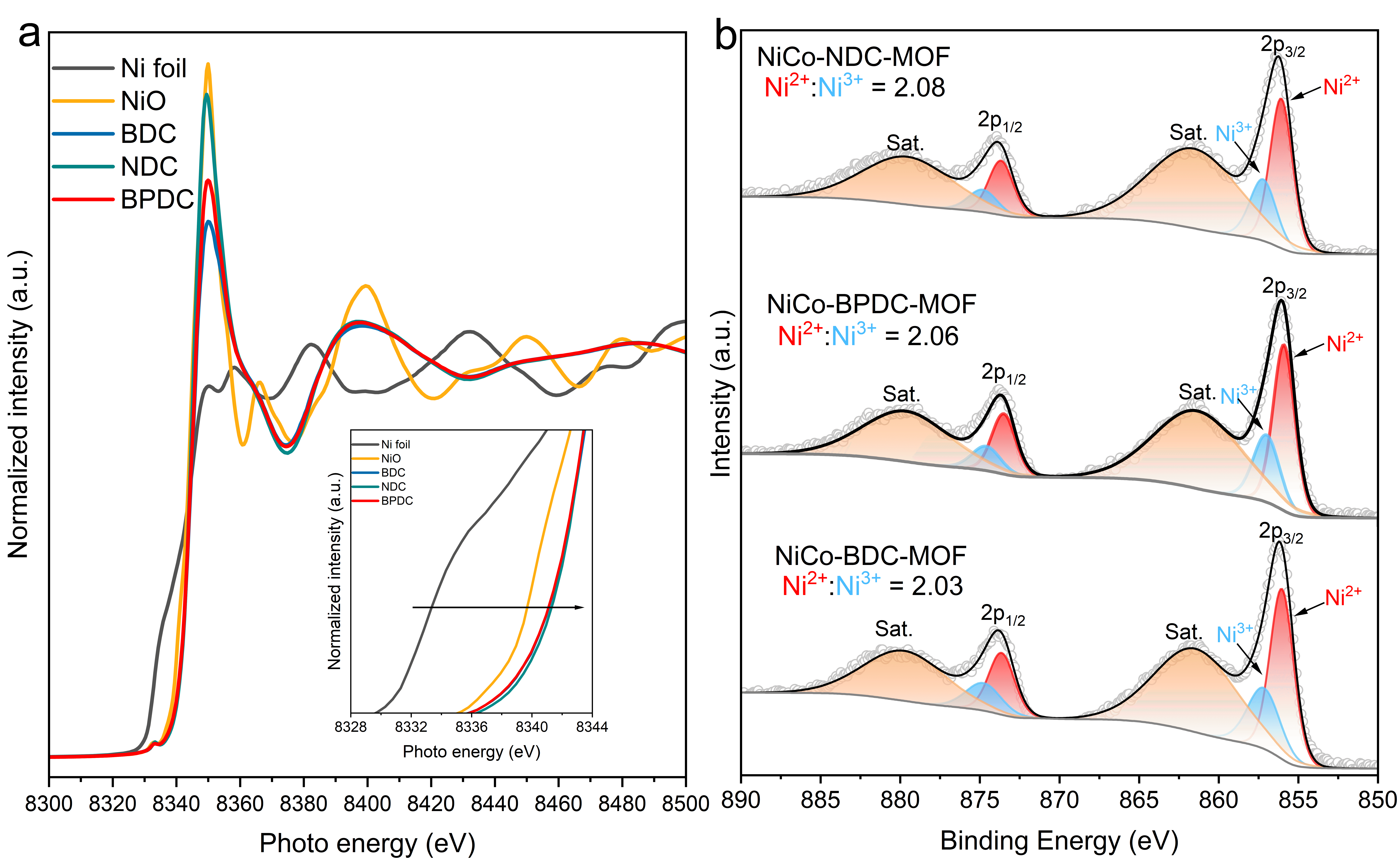


**Figure S5**. (a) Ni K-edge XANES spectra. *Inset*, the magnified pre-edge XANES region. (b) Ni 2p XPS spectra.


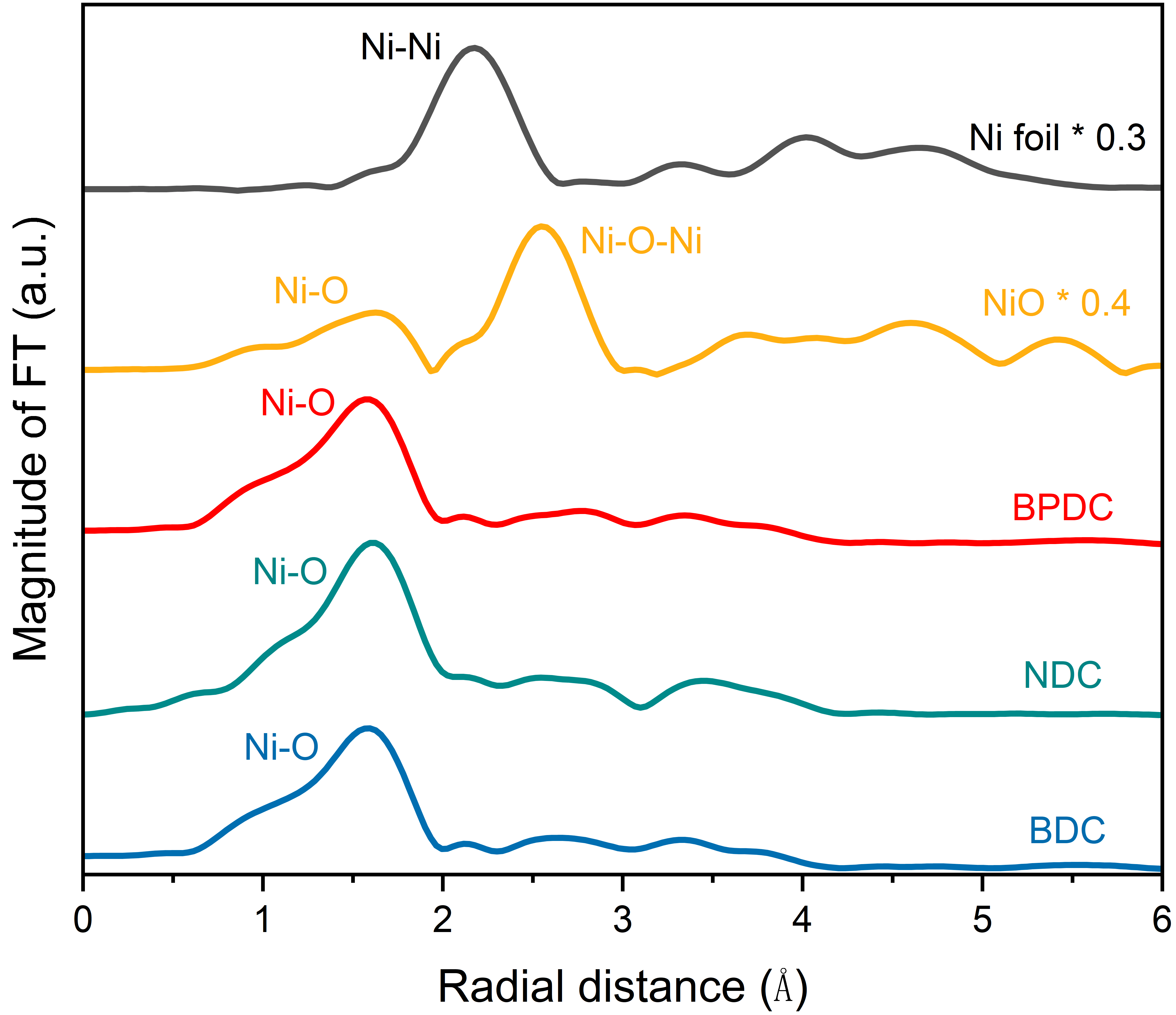


**Figure S6**. Fourier-transformed EXAFS spectra.


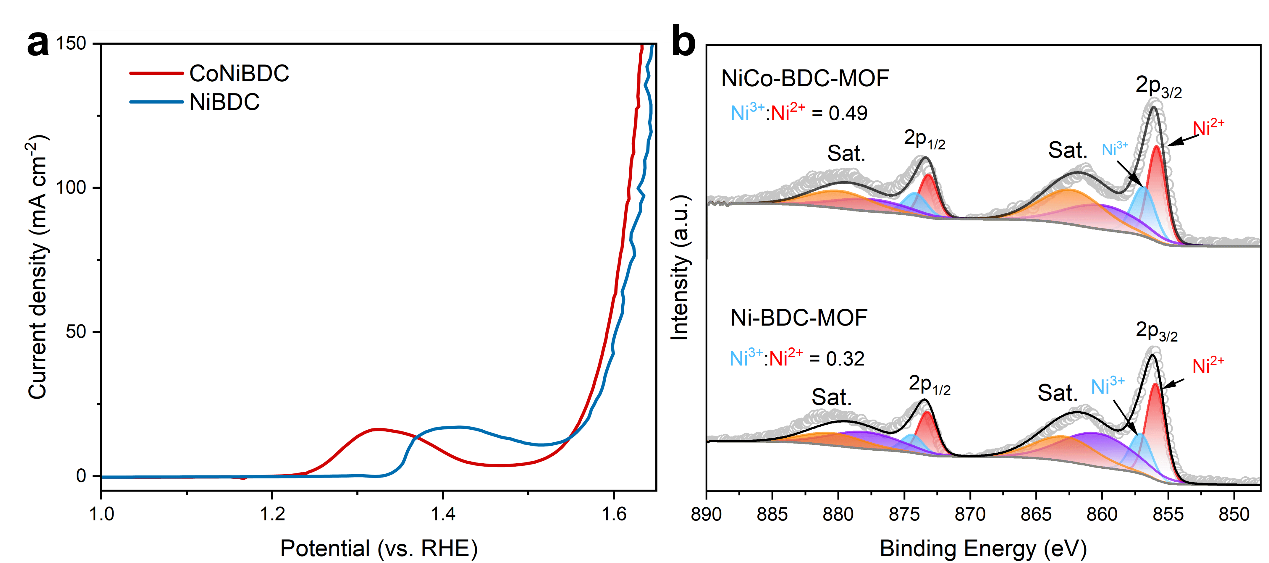


**Figure S7**. (a) LSV curves of Ni and NiCo-BDC MOF for OER. (b) Ni 2p XPS spectra of Ni and NiCo-BDC-MOF.


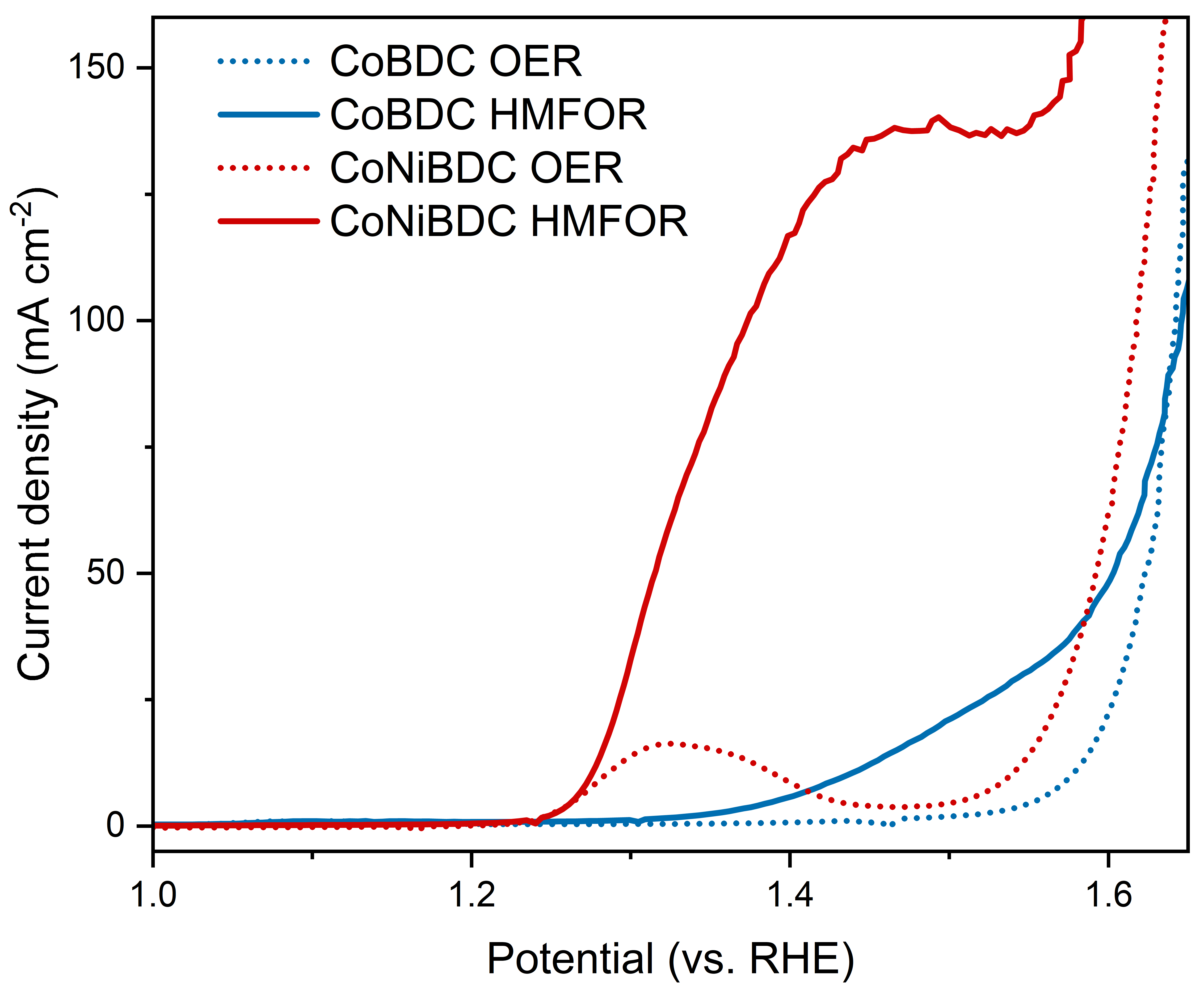


**Figure S8**. LSV curves of Co and NiCo-BDC-MOF for OER and HMFOR.


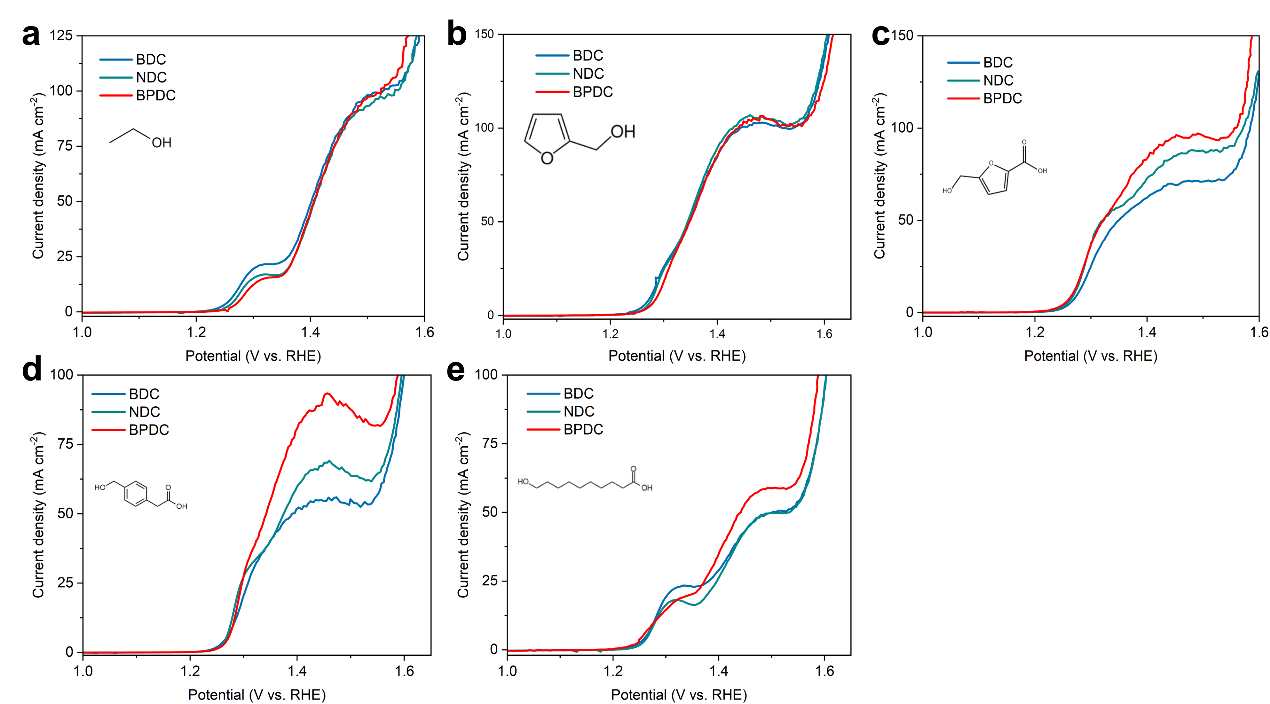


**Figure S9**. LSV curves for (a) ethanol, (b) furfuryl alcohol, (c) 5-hydroxymethyl-furan-2-carboxylic acid, (d) 4-(hydroxymethyl) phenylacetic acid and (e) 10-hydroxydecanoic acid.


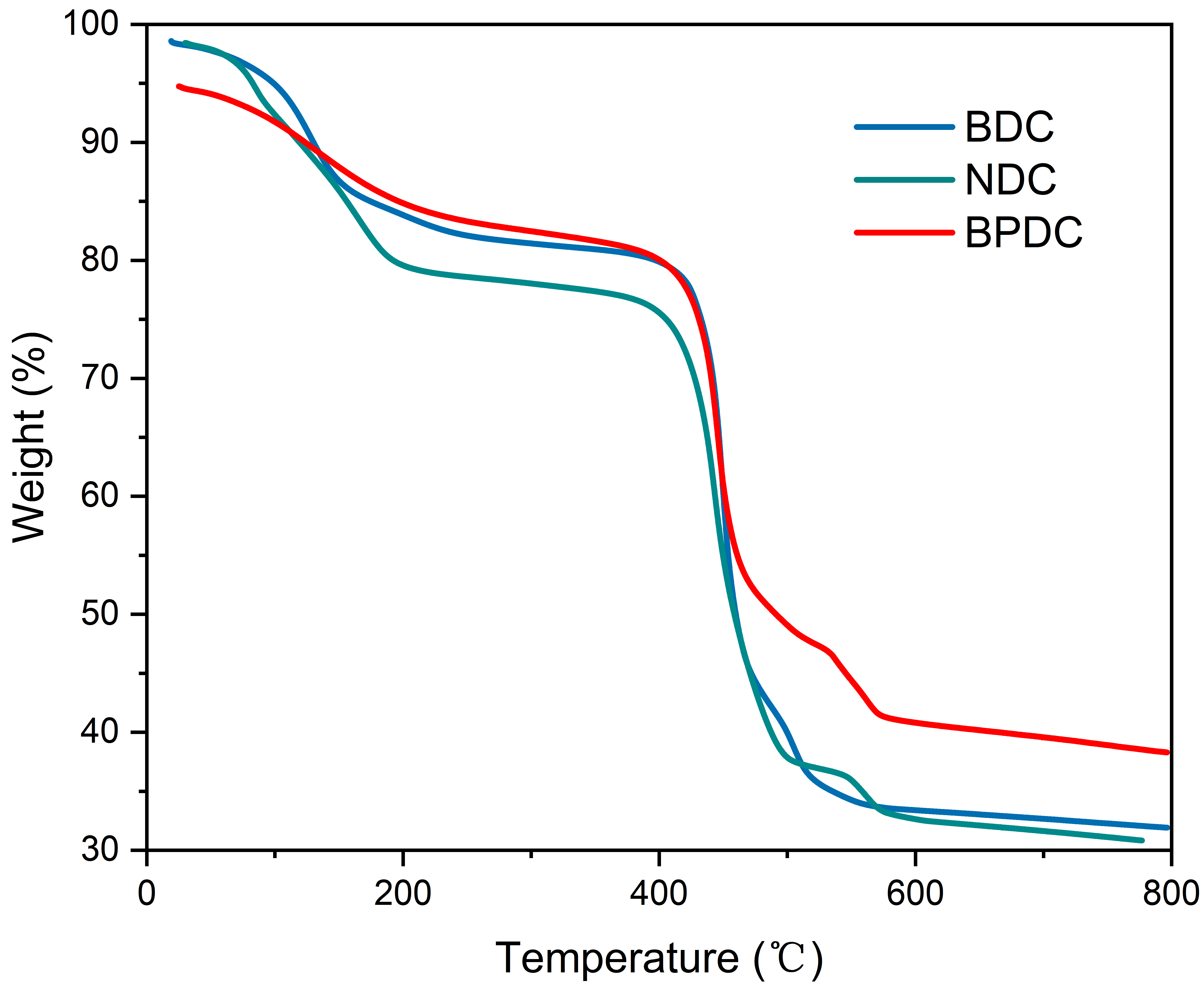


**Figure S10**. Thermo-gravimetric analysis of three MOFs.


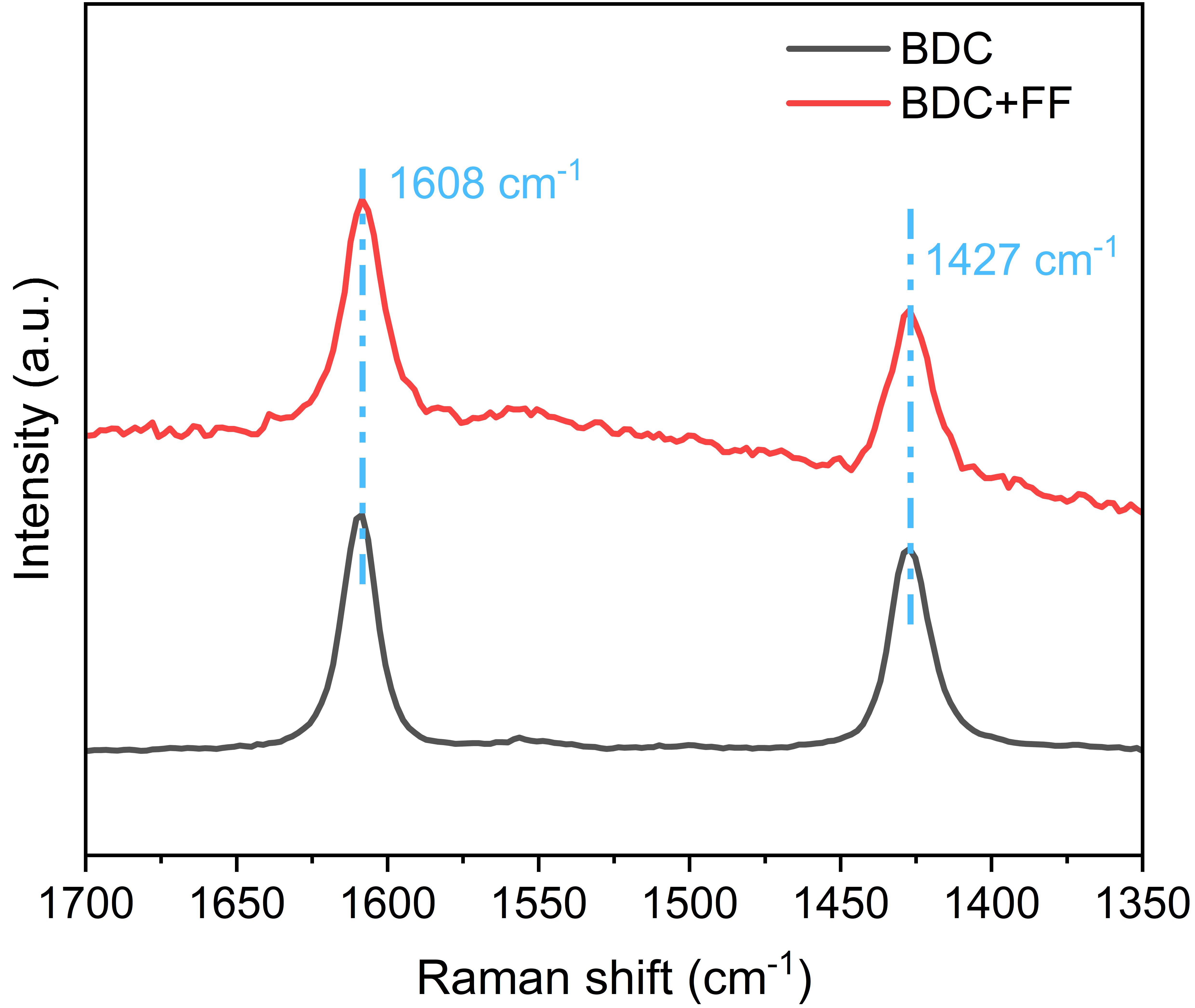


**Figure S11**. Raman spectra of BDC-MOF before and after furaldehyde adsorption.


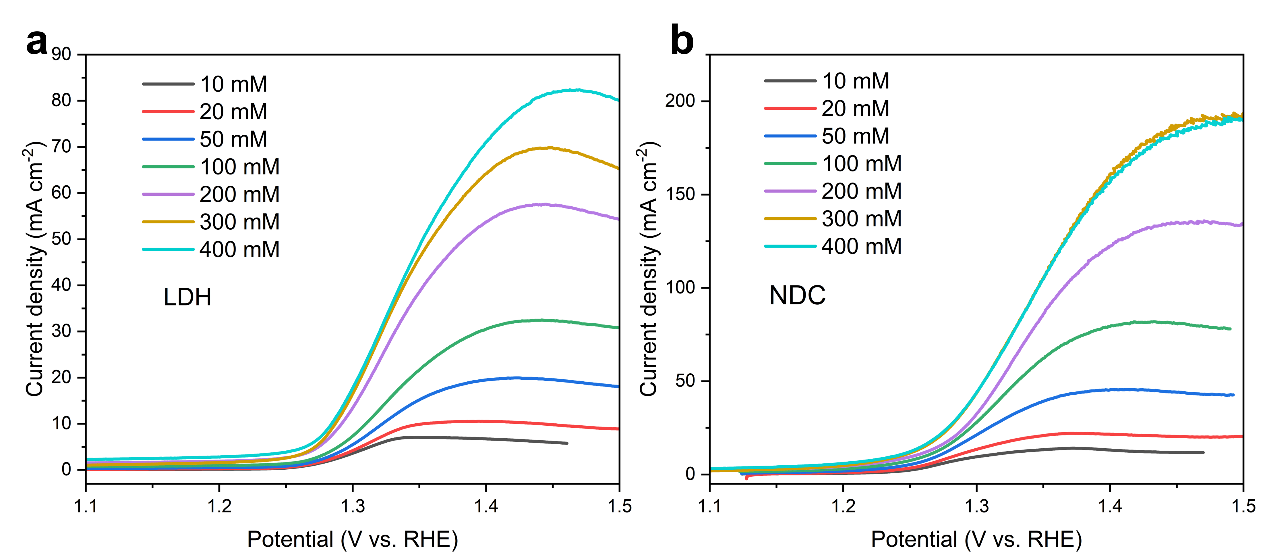


**Figure S12**. LSV curves of (a) LDH and (b) NDC at different furaldehyde concentration. To ensure accuracy of Tafel slope, the scan rate is 1 mV/s.


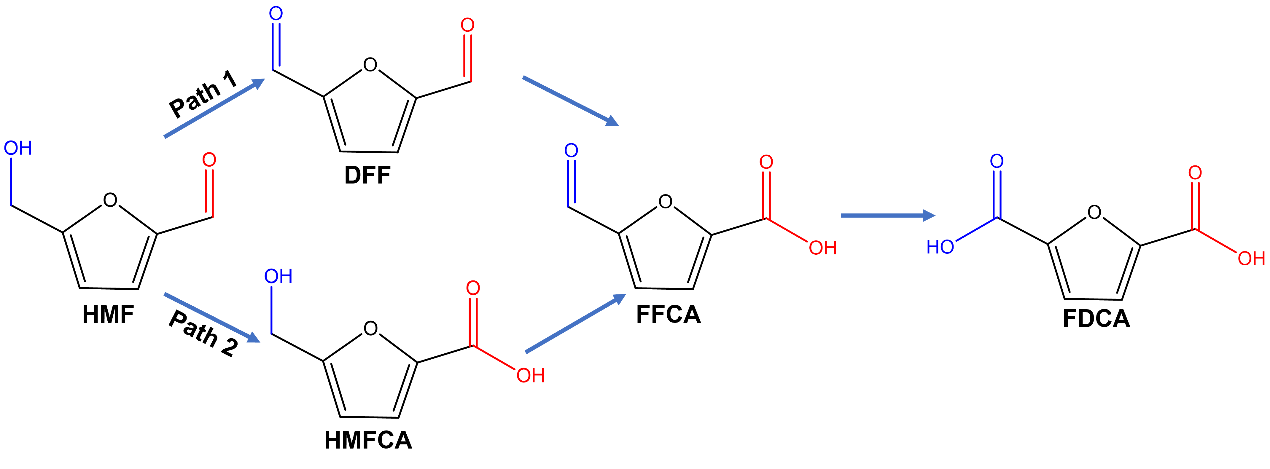
 **Figure S13**. Pathways of HMF oxidation to FDCA.





**Figure S14**. HPLC standard curve measurements of pure FDCA, HMFCA, FFCA, HMF and DFF model compounds.


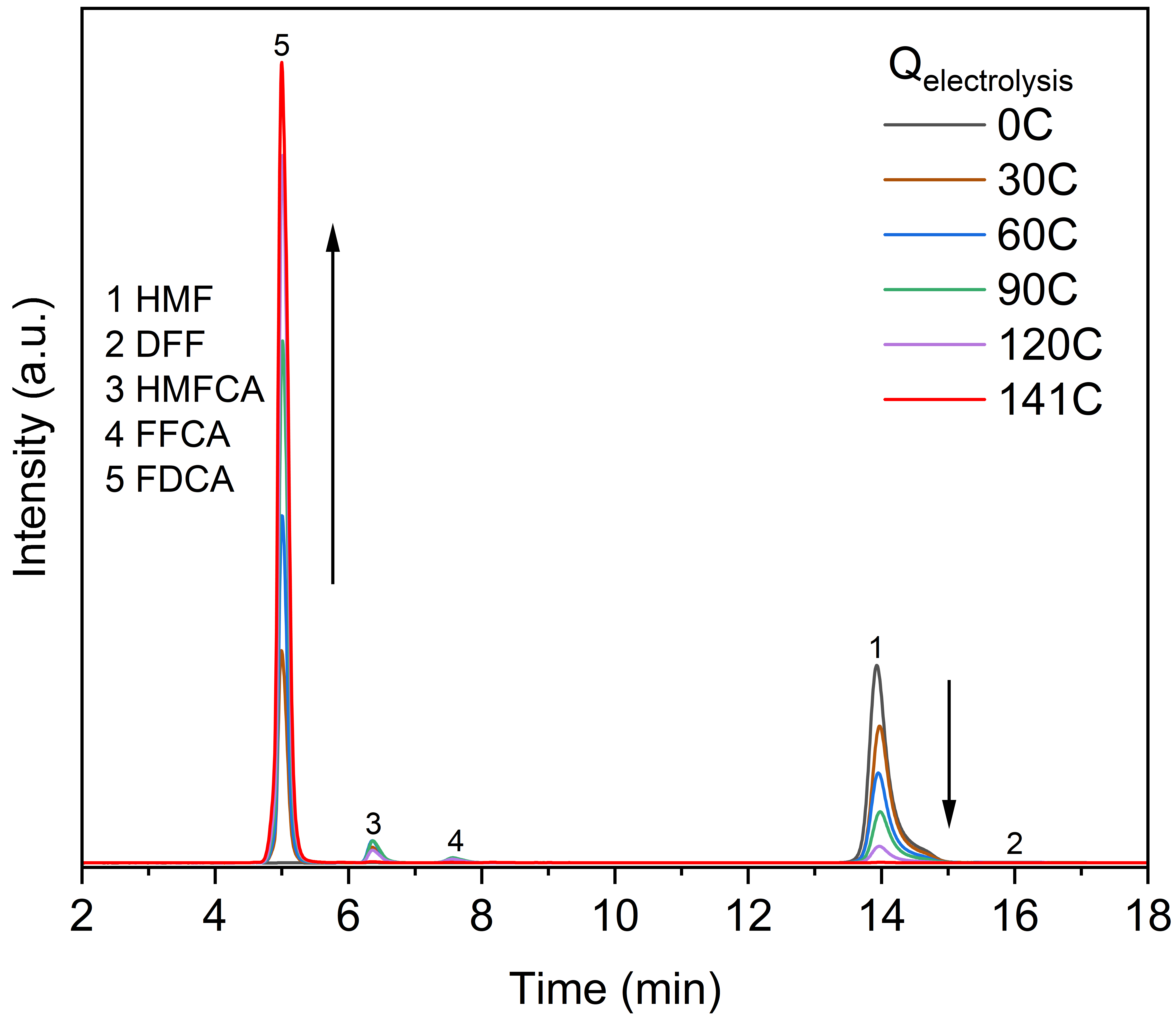


**Figure S15**. High-performance liquid chromatography chromatograms obtained at various electrolysis charges for HMFOR.
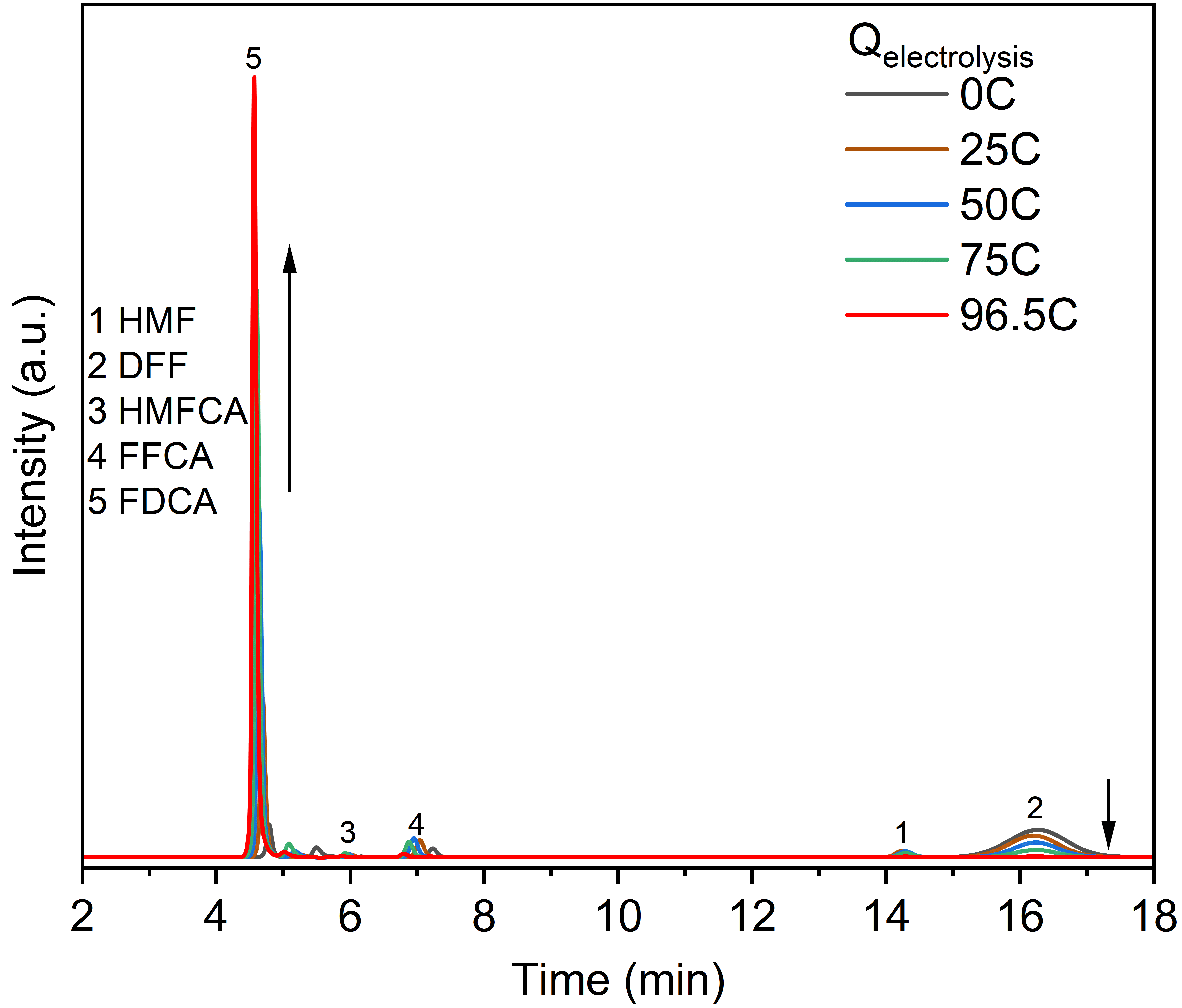
 **Figure S16**. High-performance liquid chromatography chromatograms obtained at various electrolysis charges for DFFOR.


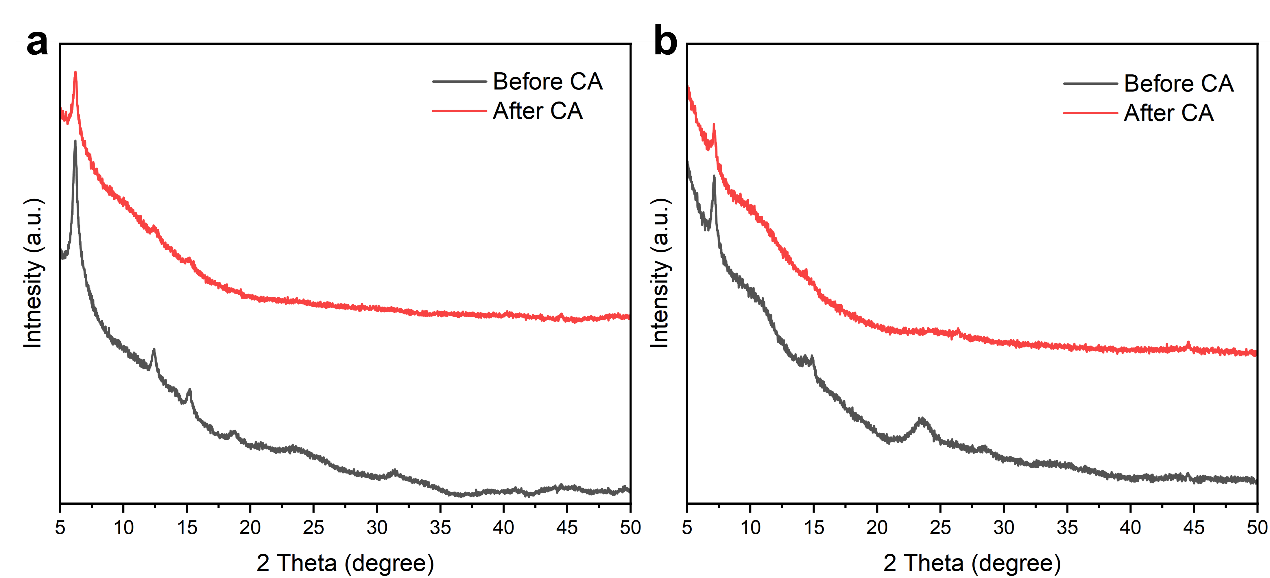


**Figure S17**. (a) XRD of BPDC-MOF before and after HMFOR CA test. (b) XRD of NDC-MOF before and after DFFOR CA test.


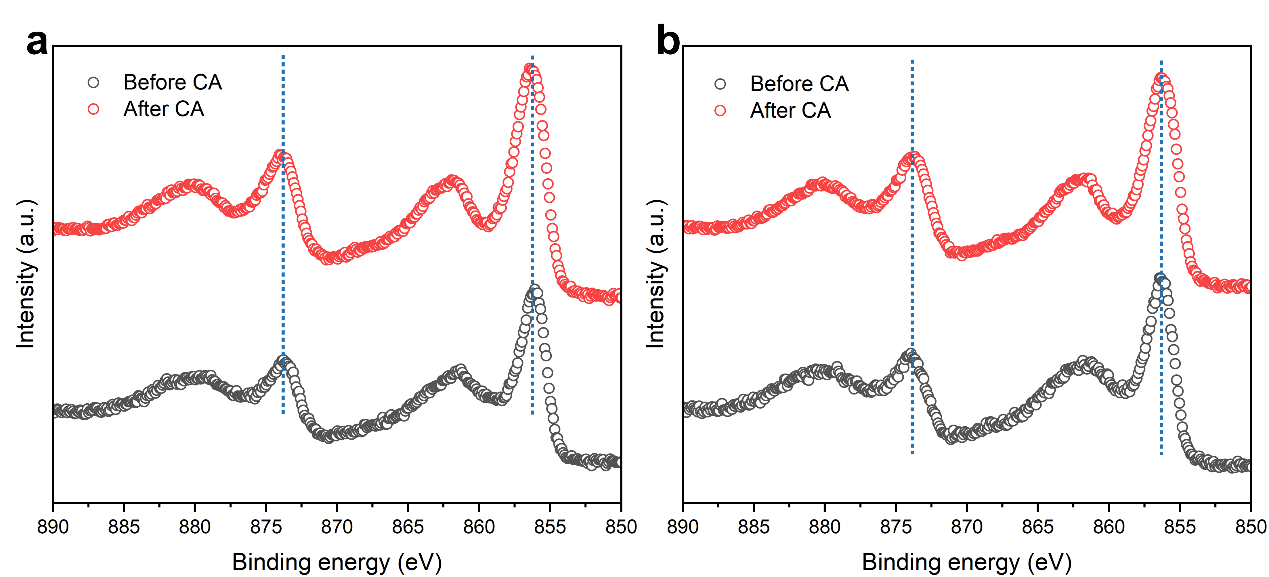


**Figure S18**. Ni 2p XPS spectra of (a) BPDC-MOF before and after HMFOR CA test. (b) NDC-MOF before and after DFFOR CA test.


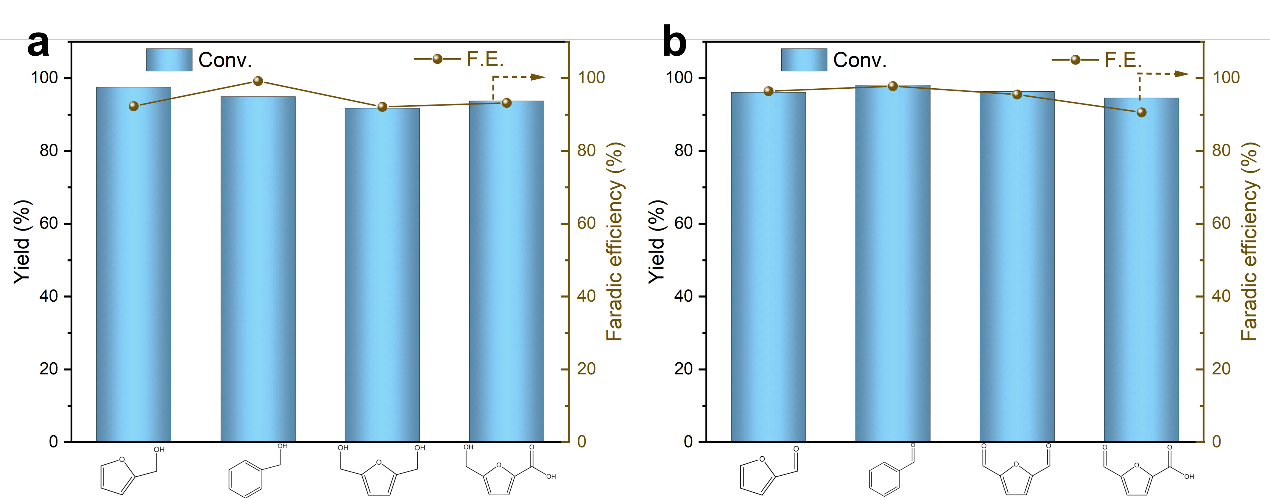


**Figure S19**. Different organic acid yield and Faradic efficiency of (a) BPDC-MOF for alcohols’ electrooxidation and (b) NDC-MOF for aldehydes’ electrooxidation.

|  | BDC | NDC | BPDC |
| --- | --- | --- | --- |
| Ni (wt %) | 16.41 | 12.73 | 12.74 |
| Co (wt %) | 8.11 | 6.35 | 6.42 |

**Table S1**. Inductively coupled plasma atomic emission spectroscopy analysis of MOFs.

**References**

1. Kresse, G.; Joubert, D., From ultrasoft pseudopotentials to the projector augmented-wave method. *Phys. Rev. B* **1999,** *59* (3), 1758-1775.

2. Blochl, P. E., Projector augmented-wave method. *Phys. Rev. B Condens. Matter.* **1994,** *50* (24), 17953-17979.

3. Hammer, B.; Hansen, L. B.; Nørskov, J. K., Improved adsorption energetics within density-functional theory using revised Perdew-Burke-Ernzerhof functionals. *Phys. Rev. B* **1999,** *59* (11), 7413-7421.

4. Monkhorst, H. J.; Pack, J. D., Special Points for Brillouin-zone Integrations. *Phys. Rev. B* **1976,** *13* (12), 5188-5192.

5. Grimme, S.; Ehrlich, S.; Goerigk, L., Effect of the damping function in dispersion corrected density functional theory. *J. Comput. Chem.* **2011,** *32* (7), 1456-1465.

6. Grimme, S.; Antony, J.; Ehrlich, S.; Krieg, H., A consistent and accurate ab initio parametrization of density functional dispersion correction (DFT-D) for the 94 elements H-Pu. *J. Chem. Phys.* **2010,** *132* (15), 154104.

7. Skulason, E.; Bligaard, T.; Gudmundsdottir, S.; Studt, F.; Rossmeisl, J.; Abild-Pedersen, F.; Vegge, T.; Jonsson, H.; Norskov, J. K., A theoretical evaluation of possible transition metal electro-catalysts for N2 reduction. *Phys. Chem. Chem. Phys.* **2012,** *14* (3), 1235-45.
